# Driver Gaze Behavior Is Different in Normal Curve Driving and when Looking at the Tangent Point

**DOI:** 10.1371/journal.pone.0135505

**Published:** 2015-08-19

**Authors:** Teemu Itkonen, Jami Pekkanen, Otto Lappi

**Affiliations:** 1 Cognitive Science Division / Traffic Research Unit, Institute of Behavioural Sciences, University of Helsinki, Helsinki, Finland; 2 Transportation Engineering, Department of Civil and Environmental Engineering, Aalto University, Aalto, Finland; University of Montreal, CANADA

## Abstract

Several steering models in the visual science literature attempt to capture the visual strategies in curve driving. Some of them are based on steering points on the future path (FP), others on tangent points (TP). It is, however, challenging to differentiate between the models’ predictions in real–world contexts. Analysis of optokinetic nystagmus (OKN) parameters is one useful measure, as the different strategies predict measurably different OKN patterns. Here, we directly test this prediction by asking drivers to either a) “drive as they normally would” or b) to “look at the TP”. The design of the experiment is similar to a previous study by Kandil et al., but uses more sophisticated methods of eye–movement analysis. We find that the eye-movement patterns in the “normal” condition are indeed markedly different from the “tp” condition, and consistent with drivers looking at waypoints on the future path. This is the case for both overall fixation distribution, as well as the more informative fixation–by–fixation analysis of OKN. We find that the horizontal gaze speed during OKN corresponds well to the quantitative prediction of the future path models. The results also definitively rule out the alternative explanation that the OKN is produced by an involuntary reflex even while the driver is “trying” to look at the TP. The results are discussed in terms of the sequential organization of curve driving.

## Introduction

**Fig 1:**
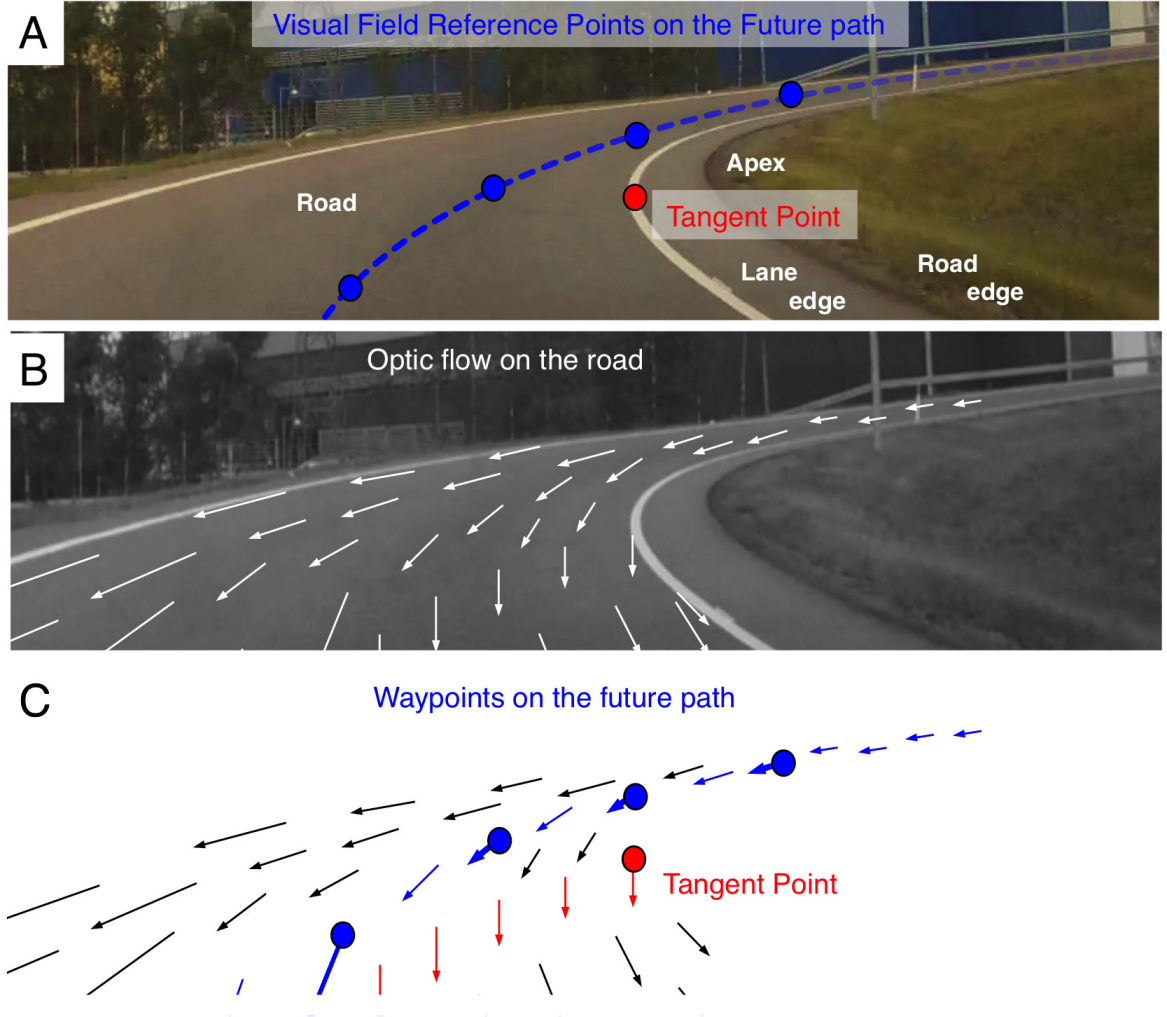
Gaze targets and optic flow when cornering in in a right–hand bend (Bend 2 of the present study). *1A*. The tangent point (TP) is a point where the line of sight is tangential to the lane edge (which is why this point is called the tangent point), at the tip of the apex. It is the point where the orientation of the projection of the edge line in the driver’s visual field is reversed. The future path (FP, dotted blue line) corresponds to the vehicle trajectory at future points in time, and has no distinctive visual features to mark it. *1B*. Visual flow on the road surface. *1C*. Visual flow at the reference points in 1A. Note the presence of horizontal flow at the FP points, and purely vertical flow at TP.

This study investigates eye–movement patterns and gaze behavior in bends (in normal driving, on a real road). Several steering models in the visual science literature attempt to capture the visual strategies used in driving. One of the main points of contention has been whether the driver uses a small number of *steering points* to determine appropriate steering, and, if so, whether these steering points are points on the *future path* [1–7]–i.e. points on the road surface they desire their trajectory to fall on–or whether *tangent points* [8–11] are used instead of or in addition to future path steering points (Fig 1).

It has been difficult to differentiate between the models’ predictions in real-world contexts, and so most of the real world data collected in the past 20 years has been ambiguous and failed to resolve the issues (for a review of the relevant models and on-road literature, see [12]). Here, an experimental manipulation was used wherein drivers are either a) instructed to either “drive as they normally would” (“normal” condition), or b) to look at the tangent point (“tp” condition).

Kandil et al. [13] used a similar experimental design (different gaze instructions) to differentiate between the tangent point and the future path as gaze target. Subjects were first told to drive as they would normally, and with no restrictions on spontaneous gaze behavior (corresponding to our “normal” condition). Then, in a second phase of their experiment, drivers were told to either look at the tangent point–i.e. to maintain permanent fixation on TP (we will refer to this below as the “tp” condition)–or use a waypoint strategy. This was done by instructing subjects to “successively look for and keep fixating for several seconds at points on the future path of the car". They called this the “gaze sampling” condition (for reasons explained below, we did not use this type of instruction in the present study).

Kandil et al. [13] report less variability in the steering signal in the tangent point condition compared to the gaze sampling condition. They interpret this “smoother” and “more stable” steering as evidence *for* the tangent point models, and *against* future path models. There are, however, several problems with this conclusion. First, their measures of “stability” and “smoothness” are based on the (questionable) assumption that the optimal driving line is always to maintain a central lane position throughout the bend, and any deviation from this ideal line is therefore steering error. Second, the analysis of eye–movements was done by hand, simply binning fixations into AOIs. AOI binning is not a very useful method for differentiating between the future path and the tangent point models (see [12],[14]). Doing this by hand, furthermore, adds a subjective element. Also–as the authors themselves note–the normal pattern in the “normal” condition was *not* to continuously “stare” at a single point on the future path (nor at the TP). Thus, the "gaze sampling" instruction they used to contrast with the “TP” instruction required a highly artificial kind of gaze behavior *not* predicted by the future path models. Finally, the authors merely state that they did not “see” evidence for gaze sampling (tracking fixations) in the “normal” condition, but no data or quantitative analysis is presented.

In [15] and [16] we have been developing a new methodological approach to assess eye–movement behavior in driving–one that is better suited for quantitative comparison of the TP and FP models’ predictions. It is based on using *optokinetic nystagmus eye–movements* (OKN) as a means for differentiating between the models.

OKN is a periodic small-amplitude movement of the eye characterized by alternating slow phase (SP, resembling a smooth pursuit or an optokinetic reflex) and a re–setting quick phase (QP, a saccadic, rapid eye movement) movements. For example, looking out of the window of a moving train elicits this type of oculomotor pattern. Also a fixation *tracking* physical points in the 3D scene–such as a waypoint on the road while driving–will cause a pursuit following the optic flow at the point of fixation. If the tracking fixation is then followed by fixation of another reference point further up the road, this will also create the characteristic SP/QP nystagmus pattern [12],[15],[16]. Note that in the latter case, a coherent *global* pattern of retinal motion is not present during curvilinear motion ([3],[17]; Fig 1B). We will nevertheless call the SP pursuit movement “optokinetic”, as it follows *local* optic flow. We do this without committing to any hypothesis concerning the underlying neural mechanisms–that is, whether it results from actively tracking a focal visual target in 3D, or whether it is reflexively elicited by retinal image slip. We use the term OKN in a purely descriptive way.

**Fig 2:**
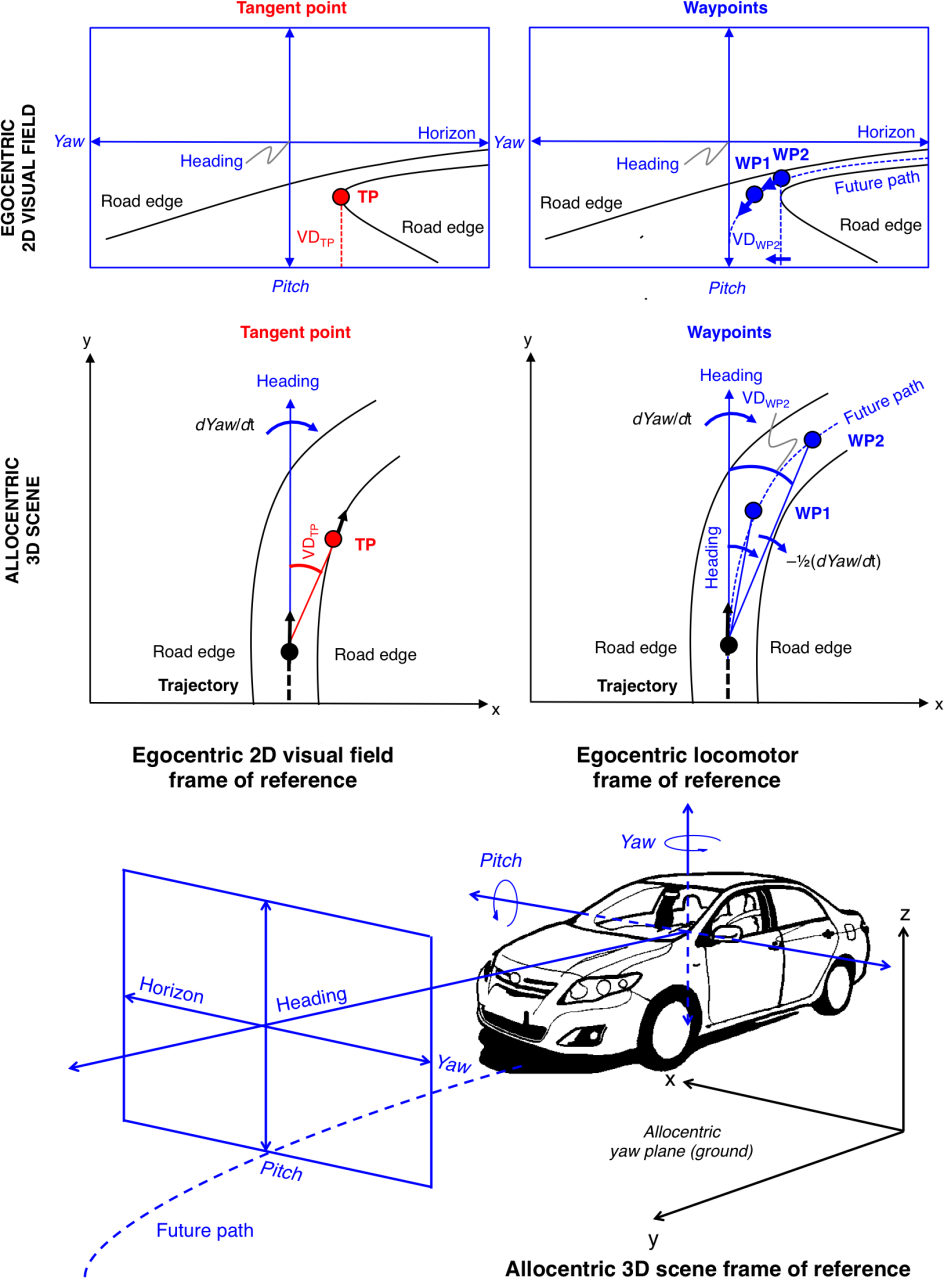
Movement of the reference points in the visual field and in the allocentric scene. *Top left*. During constant radius cornering, the inside lane edge presents a static feature in the visual field, the visual direction of the tangent point (angle relative to heading) does not change. *Middle left*. The tangent point is a travel point: it moves in the allocentric scene as the observer moves on the curved trajectory. *Top right*. Waypoints on the future path follow the local visual flow: in the visual field they move from an initially eccentric position towards the direction of heading. *Middle right*. In the allocentric scene waypoints are at rest. As vehicle heading rotates in the yaw plane, the lines of sight to the waypoints rotate at ½ yaw rate (this applies to constant–radius circular path only). *Bottom*. How the egocentric and allocentric frames of reference are related. The locomotor frame of reference and the driver’s visual field are both egocentric frames, referenced to heading (current direction of locomotion).

OKN has been shown to be present in real ([15],[16]) and simulated ([18]) curve driving. The key feature relevant for the present study is that the SP follows local optic flow at or around the point of regard, stabilizing the local retinal image in the foveal region. For a point on the future path in the far zone (FP, Fig 1C), this flow will be both horizontal in the direction opposite to the direction of the bend, and vertical downwards (cf. Figs 1B, 1C and 2A). Looking at Fig 1C we can see that while the TP and the FP waypoints nearest to it are very close to one another in the visual field–and therefore difficult to resolve in noisy real–world data–*the visual flow at these points is quite different*. The flow at TP being vertical ([8]), whereas the flow on the future path has a definite horizontal component ([3],[17])

Analysis of OKN thus offers a way of contrasting predictions from the tangent point and future path models. It overcomes some of the limitations of traditional area of interest (AOI) methods which have proved inconclusive in differentiating between FP and TP models in on-road studies ([12]). In this study, we therefore set out to investigate the differences in the strategies through *a similar experimental manipulation* to [13], but using the *more powerful objective gaze behavior analysis methods* developed in [16].

Both TP and FP models are based on observing visual features that fall into a fairly small part of the visual field and thus the predicted *gaze distribution* is very similar, and noise in gaze position measurement makes traditional AOI methods inconclusive. This makes it methodologically challenging to differentiate between the models in naturalistic real driving. The present argument is based around the hypothesis that *the different strategies will predict different OKN patterns*. To see how this comes about, we need to discuss the hypotheses in a bit more detail.

### Derivation of the OKN eye–movement predictions from future path and tangent point models

Different FP models disagree in terms of whether the target points on the future path are waypoints (fixed in the 3D scene) or travel points (traveling with the observer), and waypoint models differ in terms of where and how far ahead waypoints on the future path might be. All waypoint models, however, predict optokinetic nystagmus in the driver's eye–movement pattern (for review, see [12]). What is more, a definite prediction on the horizontal *angular velocity* of the OKN SP can be made: on a circular trajectory, the magnitude of the optic flow horizontal component is ½ of the rate of rotation in yaw axis. So, if a waypoint on the future path is fixated there should be a close correspondence between the horizontal speed of the OKN slow phase and vehicle yaw rate ([3], [17]). To get an intuitive grasp of this prediction, one can imagine an observer driving a vehicle on a circular arc that spans precisely 90°. If the observer would place himself to one end of this arc, and fixate the end of his path (at a distance of 1/4 of the circumference of the circle, along the path), then the angle between his instantaneous heading and his gaze target would be 45°. By turning the vehicle so as to always keep it tangential to the radius of the circle, the observer would have rotated by 90° when the end of the arc is reached. Meanwhile if he or she has maintained fixation on the endpoint, the angle between that point and the vehicle's heading would have fully closed, which means they eyes will have rotated by 45°, half the amount of observer rotation. This same 2:1 ratio holds regardless of which point on the arc one decides to fixate, which means that under perfect tracking the eyes will counter-rotate by half the rotation rate of the observer.

If the driver is fixating the TP, then what is the prediction for OKN eye movements? The tangent point is stationary in the visual field, but it is *not* fixed in the 3D scene. Instead, it moves along with observer motion (cf. Fig 2)–it is a *travel point*, not a *waypoint* [12]. In a circular bend, a trajectory with constant lateral lane position corresponds to a state where the visual angle between heading and the line-of-sight to the tangent point (visual direction, VD) remains constant. In this case, also the visual flow at the tangent point is vertical [8]. Thus any movement in the tangent point visual direction or–equivalently, deviation of flow at the tangent point from the allocentric vertical–would indicate a change in path curvature or bend curvature (steering error), to be compensated. Thus, the tangent point is not predicted to move horizontally in the visual field–except for small movements (in either direction), reflecting steering error–and therefore gaze is not expected to present a systematic horizontal OKN.

As for the vertical movement, this depends in part on one’s assumptions about the tracking mechanism. We may consider two alternatives: (1) If the fixation is “perfect”, then there will be no OKN at all, instead, the gaze will stay at the TP, and any (non–fixational, tracking) eye movements will be small, following the movement of the tangent point, should steering errors and corrections occur. (2) However, fixation may not always be in “perfect” in the above sense. The optic flow around the TP direction may elicit an involuntary *optokinetic reflex* (OKR) “dragging” gaze from the tangent point, followed by a correcting saccade. This might, conceivably, induce OKN even when looking at the TP. In the case of the complex flow field in curve driving, however, there will be no *globally* coherent flow. What direction and magnitude the OKN SP will then take depends on the way the OKR integrates retinal slip. As said, the local flow *at* the tangent point is vertical (in ideal conditions), on which basis one might predict a vertical (downwards) SP. On the other hand, flow in the tangent point *region* is variable, and differs greatly below and above the tangent point.

## Aims of the Study

It was shown in [15] and [16] that the horizontal movement of the eye during the OKN SP is in agreement with what one would predict if the driver is tracking waypoints on their future path (*OKN slow phase horizontal angular velocity approximately ½ vehicle yaw rate*). On geometrical and physiological grounds the TP hypothesis would by *default* predict no (horizontal) optokinesis (*perfect TP fixation*). However, auxiliary assumptions about eye–movement reflexively following local optic flow at or around the tangent point (*TP fixation + “noise” from unsuppressed OKR*) could accommodate different eye-movement patterns into a tangent point model. This alternative explanation–while admittedly somewhat *post–hoc*–could not be ruled out in [16]. The naturalistic task (only to drive as normal) used in that study meant that we could not directly test whether the different gaze strategies really produce measurably different eye–movement patterns.

The main aim of the present study is *to check the validity of the assumption that looking at the TP does indeed lead to a measurably different gaze pattern from the OKN observed in natural driving*. The present experiment therefore acts as a control for the “TP + ‘OKR noise’” interpretation of the data in [16].

Also–by using the data from that experiment as a control group–we will be able show empirically that the gaze behavior observed in the “normal” condition (driving as normal) in the new experiment itself corresponds to natural driving (i.e. interleaving “normal” driving with “tp”–instruction to keep looking at the TP–does not in itself affect the “normal” pattern).

## Methods

### Subjects

Ethical permission for the research was granted by the Ethics committee of the Faculty of Behavioural sciences, University of Helsinki.

21 subjects participated in Experiment 1 (reported in Lappi et al., 2013b, 11 male, age range 22–49 y, mean 27 y), and 10 in Experiment 2 (all male, age range 24–35 y, mean 30 y). Requirements for participation were normal or corrected vision and valid a driver's licence. For participants with corrected vision, only contact lenses were allowed, to minimize any interference that eye glasses could cause to the eye tracker signal. Participants were recruited through university mailing lists, advertising for “experienced drivers”. The subjects' self-reported kilometrage varied from 1 000 to 1 000 000 km (Experiment 1), and 10 000 to 300 000 km (Experiment 2).

### Equipment

The instrumented car was a model year 2007 Toyota Corolla 1.6 compact sedan with a manual transmission. The car was equipped with a two-camera video–based remote eye–tracker (Smart Eye Pro version 5.5 (Experiment 1) 5.7 (Experiment 2), www.smarteye.se) operating at 60 Hz. A forward facing VGA scene camera captured the front view at five frames per second. Vehicle speed, yaw rate and control signals, such as steering angle, throttle and brakes, were recorded from the CAN bus at 100 Hz, subsequently downsampled to synchronize with the eye tracker. GPS position (without differential correction) was recorded at 1 Hz. A computer, located in the rear luggage compartment and running custom MATLAB software, synchronized and time-stamped the data during the procedure.

### Route

**Fig 3:**
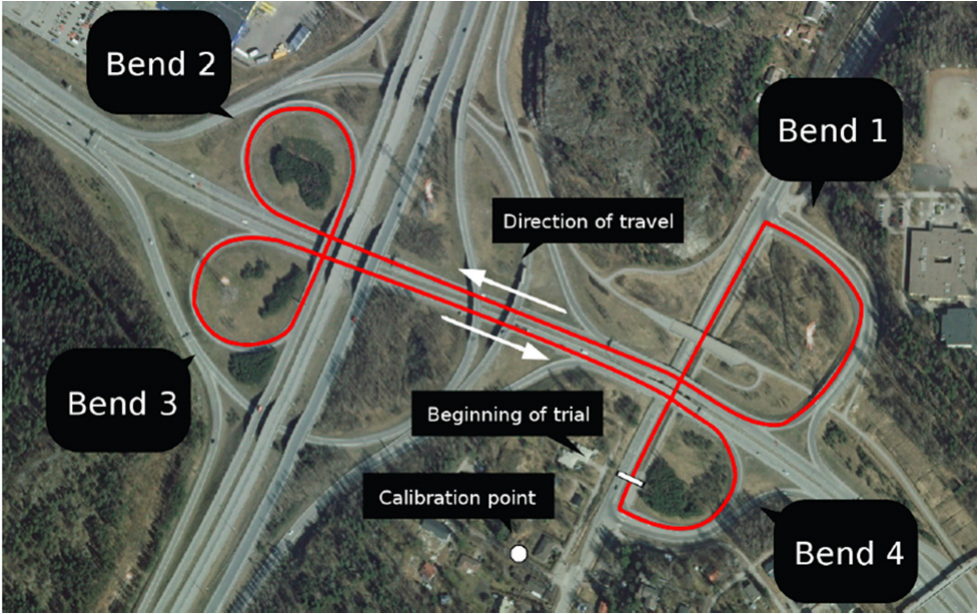
Aerial image of the experiment location. Red line traces the route that the subjects drove. Calibration site was used to check the accuracy of the eye tracker in between series of trials. Beginning of trial was selected to start after bend 4, near the calibration point. Aerial image courtesy of National Land Survey of Finland, 2013. Licence: CC 4.0 http://creativecommons.org/licenses/by/4.0/deed.en

The experiments took place in the interchange of the outermost ring road in Helsinki (Kehä III) and Finnish national road 4 (E75) (60.2742°N, 25.0879°E). Both roads are dual carriageways and are connected with right-handed, steeply curving one-way ramps. A third road, some 200 m away, which intersects with Kehä III and had similar ramps, was used to turn the vehicle around and return to the motorway, so that the overall route took a loosely cloverleaf-like form (**Fig 3**). The speed limit on both motorways (and thus also the bends analyzed) was 80 km/h.

Road geometry and the lack of an opposing lane were the foremost reasons for choosing the route. The motorway ramps have a clear sustained cornering phase, where the yaw rate of the car remains relatively stable for several seconds, allowing the collection of a relatively large amount of gaze data under constant, relatively unchanging stimulus conditions. The ramps in question also had a continuous white edgeline on the right road edge, which could later be used to identify the tangent point unambiguously.

Bends 2 and 4 were analyzed in this study. They were geometrically similar in curve radius, lane width and road span, though Bend 4 did not connect to a highway at the exit and therefore had a shorter cornering phase. Also, Bend 2 had an uphill gradient and Bend 4 a downhill gradient. Bend 1 was not analyzed because of its different (variable curvature) geometry. Given that the derivation of horizontal eye–movement prediction from the FP models requires that both the car and gaze landing point need to be on a constant–curvature path, Bend 1 is not suitable. Bend 3 was not analyzed because as it joins the motorway, a lane change to join the traffic flow on Kehä III is required. This causes anticipatory glances away from the bend (to identify gaps) during cornering. This behaviour directly conflicts the instruction in the TP condition, and in conflict situations we emphasized the priority of safety, rather than following the instruction.

The experiments were conducted between morning and evening commutes or after 5 p.m. to minimize the amount of traffic at the location. The experiments were conducted driven between August and October 2011 (Experiment 1) and in July 2013 (Experiment 2).

### Procedure

Upon arrival, the subjects signed informed consent, and filled in a questionnaire on driving background, including self-reported annual and lifetime kilometrage.The route was shown on a map, and the general purpose of the study (to measure eye movements while driving) was explained, but the subjects were kept naïve about the specific hypotheses (tangent point vs. future path). Approval to conduct the research was obtained from the ethics committee of the Faculty of Behavioural Sciences, University of Helsinki. Written informed consent to participate in this study was obtained from each participant. This was done, in accordance with the approval of the ethics committee, in the form of a fixed-format consent form explaining the purpose of the study, the procedure, and intended use of the data (for scientific purposes only). Paper copies of the consent forms were archived.

Subjects drove the route a total of 16 times. On the first trials, the subjects were instructed about which lanes and off-ramps they should take, after which they drove independently. Otherwise conversation was kept to a minimum during the runs.

In Experiment 1, subjects were instructed to drive normally and told that no additional tasks were required of them. In Experiment 2 the “laps” around the interchange were equally divided into two conditions: The “normal” condition, acting as control, had the same instruction as Experiment 1. The “tp” condition introduced an additional condition where the subjects were asked to locate the tangent point as soon as it became visible during the approach, and to fixate it as accurately as they could.

To minimize possible interference induced by the instruction, the experiment began with a block of “normal” runs, followed by a stop at the calibration site where the subjects were introduced to the concept of tangent point. They were then asked to verify their understanding by marking the point in photographs taken at various distances from the TP. The next block was then performed under the new instruction. In total there were four blocks which alternated between the conditions, ie. “normal”—“tp”—“normal”—“tp”. In Experiment 2 runs 1–4 and 9–12 were in the “normal” condition, and runs 5–8 and 13–16 in the “tp” condition.

### Calibration

The eye tracker was calibrated on university campus before driving to experiment location (Experiment 1) or in a car park near the test site (Experiment 2, top left corner in Fig 3). The calibration consisted of creating an individual profile for each subject with OEM software by Smart Eye AB. This included a “head model", based on six infared images (three for each camera), annotated with the subject's facial features. This provides a basis for head tracking, after which the head and eye movements are matched to the position of objects in the dashboard, such as the two infrared cameras, LED lights and a metal rod made specifically for calibration. The final step ties the eye position in head/vehicle to the outside world, and was performed by the subject looking at nine predefined points in the scene. Calibration accuracy was verified visually, and if gaze position deviated from any of the nine points (for more than around two degrees), the equipment was recalibrated.

Calibration was checked (and in Experiment 2 the “tp” instruction given) at the calibration site. The checks were in place to evaluate the accuracy of the eye–tracking signal due to mechanical vibration of the cameras. A recalibration of the gaze to dashboard objects was performed if signal quality visibly deteriorated.

### Postprocessing

After the experiment the data was handled with custom Python scripts written in the Traffic Research Unit.

Each driver's performance was split into separate trials that corresponded to one “lap" in the cloverleaf-like intersection. Then each trial was given a location-based representation by treating one of the trials as a “prototype" and matching the GPS data of all other trials to the GPS signal from the prototype trial. In this procedure, each observation obtained a parameter of distance (in meters) from beginning of trial, which was commensurable across trials and subjects. The beginning was placed right after the location where the calibration checks were performed.

Once the location-based representation was obtained, the cloverleaf-like interchange was segmented into four bends. The bends were numbered in driving order starting from the calibration point as seen in Fig 3.

Bends were further divided to approach, entry, and cornering segments.

**Fig 4:**
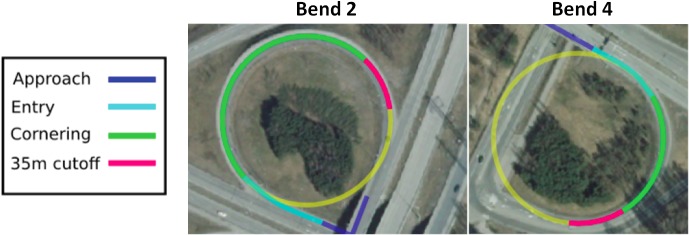
Segmentation of the analyzed bends. Approach, Entry, and Cornering phases were identified based on visually identifying the change in bend curvature from the aerial image. Note that the cornering phase of bend 2 is about twice the length of bend 4. The yellow arcs that complete the circles are included to indicate that the cornering section adheres to constant curvature. The curve radii are approximately 48m.

The segmentation of bends used in this paper differs slightly from the one used by Lappi et al. [16] (for Bend 2, which was used in that study), which used vehicle yaw rate variation as measured from the CAN bus. Note that segmentation based on road geometry abstracts away individual differences in yaw rate. The *cornering* phase was defined to be the part where road geometry forms an arc of a circle, i.e. section of constant curvature. Identification was done visually from a high–resolution aerial photograph (Fig 4). The length of the segment was reduced by cutting off 35 meters towards the exit, since both the driver and the gaze landing point need to be inside the cornering section for the assumptions of the visual steering models to apply. 35 meters was chosen as the cutoff point because it roughly corresponds to the distance to which the car would have travelled in three seconds time (with the observed mean velocity of 42 km/h, SD 3.95 km/h). This reduction, therefore, ought to give a reasonable margin for constraining unwanted effects due to other traffic and changes in road geometry between vehicle location and gaze landing point.

The *entry* segment is defined as starting 50 meters before the onset of cornering. As seen from the illustration, this segment corresponds to the section where the vehicle is turning into the bend. The *approach* segment is perhaps the most difficult to determine with precision, either by road geometry or behaviorally, and for the most part it was left out of the analysis, but included for completeness. It begins from the visual onset of the tangent point as estimated from the forward-facing video recording and precedes the entry segment directly. When the analysis at hand includes Bend 2, studied by Lappi et al. [16] the segmentation follows the one detailed above.

As a common step for all analyses, all trials with traffic, i.e. leading vehicles visible on the scene camera video, were excluded.

### Analysis of eye movements

A gaze quality criterion of 0.2 supplied by the tracker software was used to exclude data before analyses. The SEM (slow eye–movement) detection algorithm developed in the Traffic Research Unit by one of the authors (JP), and described in the appendix of [16], was applied to split the eye movement data into “fixations”. The algorithm is developed specifically to allow detection of “fixations" to targets moving (approximately) linearly relative to the observer, i.e. smooth pursuits / OKN SP movements.

The need for a detection algorithm arises because the shaking of the dashboard-mounted eye tracker results in relatively large amounts of noise in the raw signal. Calculating gaze velocities by simply differencing the signal would result in very high noise levels, and e.g. low pass filtering would mask crucial information about the signal’s structure. The present algorithm–inspired by [19] and [20] which were originally designed for general data partitioning–optimally partitions the signal into linear segments. (The underlying idea is that fixations and pursuits and saccades have approximately constant velocities, and the slope for saccades is much larger than for fixation or, in most cases, pursuit). After the segmentation was done, segments that had under 12 samples (duration 200 ms) were discarded.

The speed of each SEM was calculated by taking the start and end points of the algorithmically detected segment and fitting a straight line to the eye movement data. Then the speed was computed by dividing amplitude (difference between the calculated extremes) by duration.

Although poorly suited to differentiating between future path and tangent point models, gaze density estimate plots serve a purpose in providing an at-a-glance view of the gaze patterns. The tangent point location was used as a convenient reference point for the eye tracker signal, as it is useful for combining gaze data across subjects and trials when plotting gaze location over time. (Different people are prone to take slightly different lines, and as a result neither the TP location or future path in the vehicle's frame of reference will be exactly the same across subjects in the vehicle locomotor frame of reference). For each gaze position data point, its displacement from the tangent point position was therefore computed. Also the median displacement from tangent point was computed for each individual participant.

### Calibration accuracy

The acceptable error–used for accepting a calibration and proceeding with the experiment–was that the indicated gaze position should not be more than a couple of degrees off a designated target (during calibration, when the car and the head are immobile). A more conservative estimate of approximately 3° is more appropriate when the vehicle is on the move (for more details on the calibration method used, see the Appendix in [16]).

Lens distortion in the scene camera was corrected so that positions in image coordinates are approximately linear to the participants’ visual angles. The video frames were rectified using barrel distortion parameters estimated with a planar chessboard pattern, using OpenCV 2.4.5 [21] and pixel values for the tangent point (xy) mapped to eye-camera gaze angles (horizontal, vertical) using pinhole-model camera parameters. The TP position observation was associated with a route location by using the timestamp of each video frame.

### Tangent point identification

In Experiment 1, we had used a TP detection algorithm but for Experiment 2, the edge–line TP was manually detected from the forward-facing scene camera images. The algorithmic tangent point identification was not used because in the “tp” condition a visual marker indicating estimated gaze position in the video recording would often fall on the tangent point. This occluded the TP and made algorithmic detection unreliable. The resulting 5 Hz signal (five video frames per second) was linearly interpolated to match the 60 Hz sample rate of the eye tracker.

### Data loss

The same 17 participants’ data that were reported in [16] were analysed here (in that study, four participants’ data was disqualified because TP could not be algorithmically detected from the video image). In addition, due to rain, windshield wipers intruding on the image, or direct sunlight casting a “burned out" vertical reflection on the recording, two control subjects had Bend 3 excluded, Bend 2 was excluded from one subject, and 22 individual cornering episodes from three subjects were left out. In Experiment 2, data from three subjects were disqualified due to poor eye-tracker calibration.

## Results

**Fig 5:**
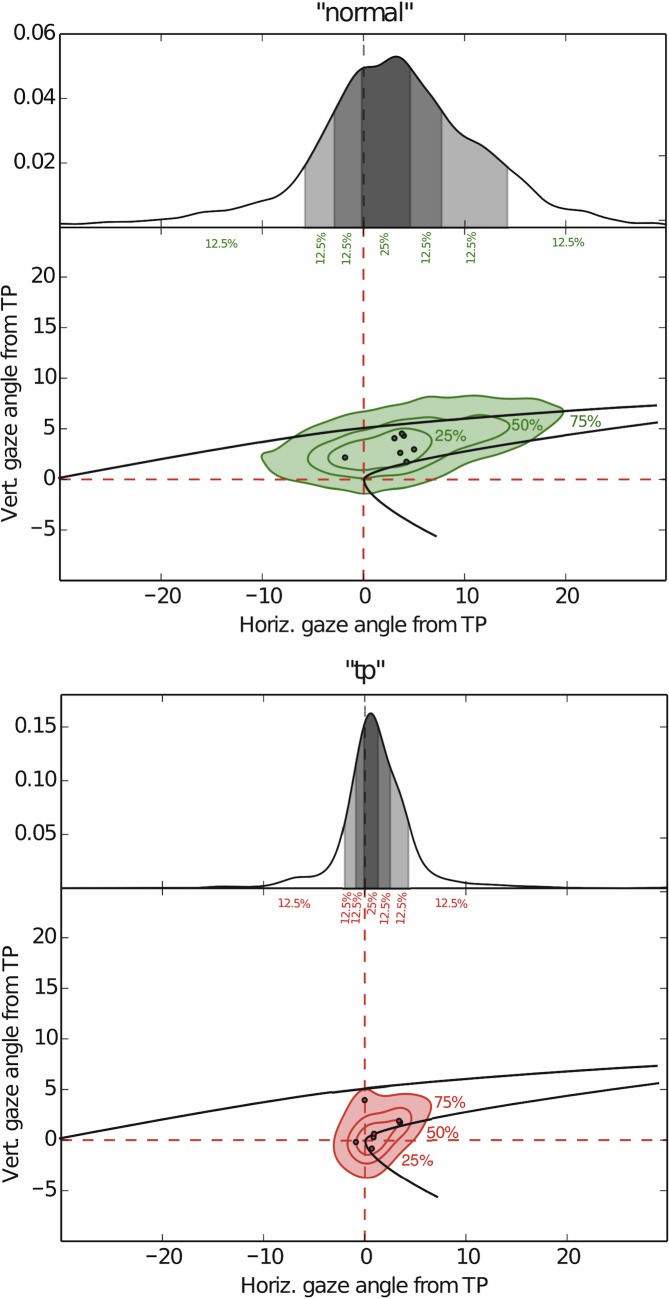
Gaze distribution in the visual field (gaze deviation from TP, TP is at the origin). *Top*. The pattern in the “normal” condition in Experiment 2 is very similar to the previously published result in Experiment 1 (Lappi, Pekkanen & Itkonen, 2013b). Gaze is widely distributed in an area, with the modal gaze position of each individual (small circles) displaced about 5° from TP. Upper panel: marginal distribution of horizontal gaze position. *Bottom*. Level curves enclose 25%, 50% and 75% of the gaze position measurements. Bottom: “tp” condition in Experiment 2. Gaze is much more concentrated, with modal gaze position well within 5° of TP.

**Fig 6:**
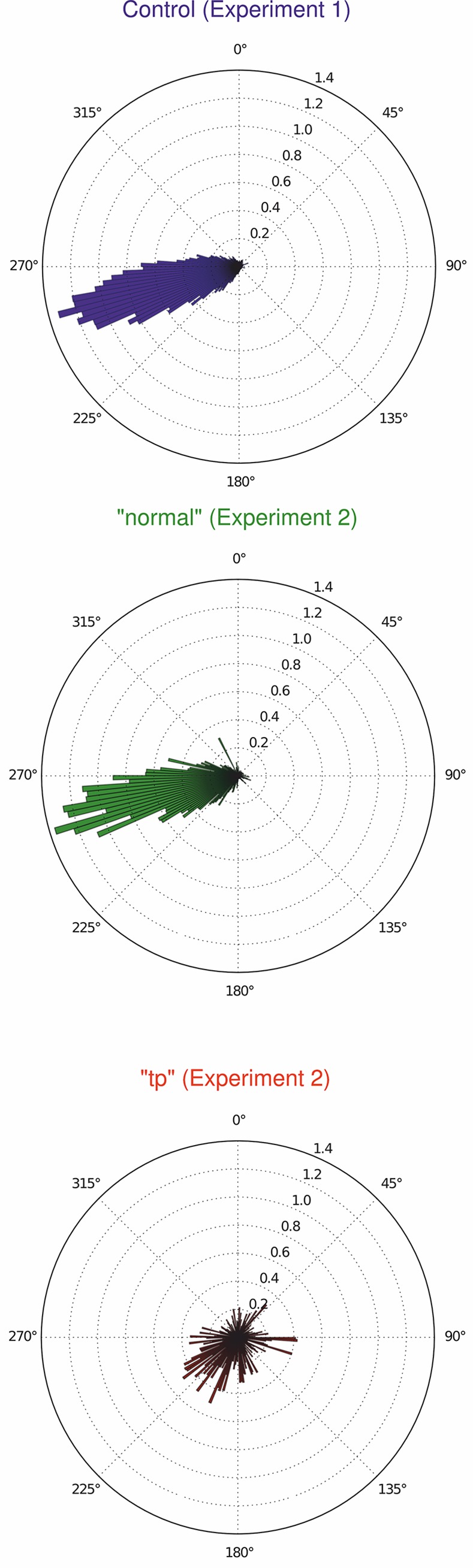
Relative frequency histogram of the distribution of the directions of identified OKN SP eye–movements (0° is vertical relative to the vehicle frame of reference, bar areas summate to unity). *Top*. Control subjects’ data (Experiment 1). *Middle*. “normal” condition (Experiment 2). *Bottom*. “tp” condition (Experiment 2). Visual pattern in the “normal” condition closely resembles Control data, and shows the distribution to be predominantly in the “to the left and slightly downwards” direction predicted by fixation tracking a point in the world. The “tp” condition pattern is markedly different, lacking this strong coherence across subjects. In each panel, the frequencies sum to unity.

Overall, in the Control and “normal” conditions gaze is concentrated above and beyond the tangent point (Fig 5). In the “tp” condition the distribution is visibly narrower, with it's locus much closer to the tangent point. The direction of the OKN SP is down and to the left in Control and “normal” conditions (Fig 6), i.e. to the general direction of the local optic flow in the far zone beyond the tangent point. This replicates the basic finding of [16], in which the Control group in Bend 2 was analyzed. In the “tp” condition, however, both the gaze–position and OKN SP direction patterns are clearly different.

**Fig 7:**
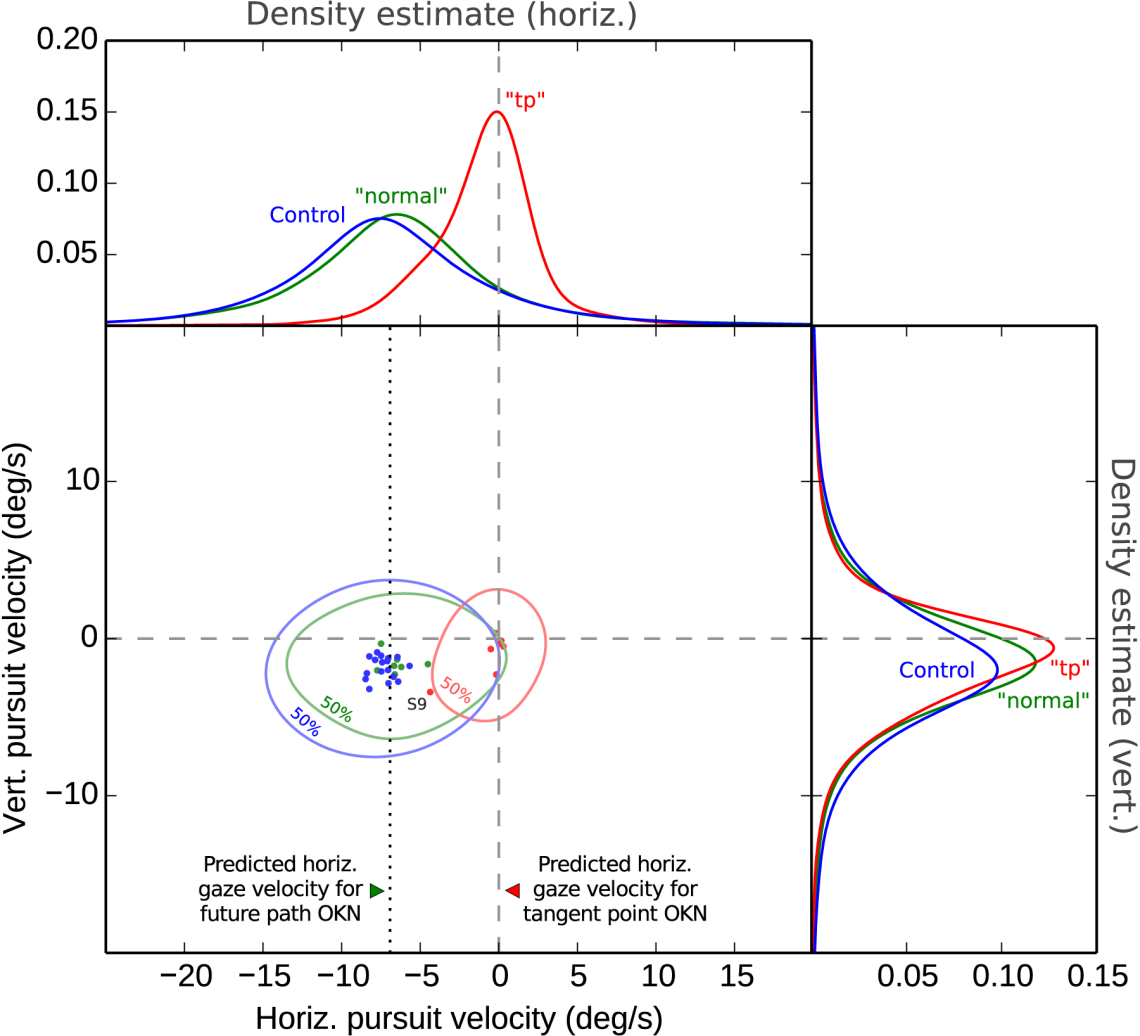
Distribution of OKN SP velocities. *Center panel*: Distribution of gaze velocities in two dimensions. Level curves enclose 50% of all OKN SP velocity measurements in the different conditions. Dots are individual participants’ mode velocities. The Control and “normal” data are clustered in the bottom left quadrant–while the the “tp” data are clustered at the zero value (except for participant 9). Dashed line indicates predicted pursuit for visually tracking a waypoint (-1/2 average yaw rate). *Top and left panels*: Horizontal and vertical gaze velocity density estimates in the different conditions.

Fig 7 shows a density estimate plot of gaze direction in 2D velocity–velocity space. The Control and “normal” conditions show gaze movement during OKN SP to be down and to the left. The direction of the gaze velocity vectors is in correspondence with the local optic flow at the future path. In the TP condition horizontal movement is clearly absent.

The intuitively most straightforward way to evaluate the dependence of OKN SP on yaw rate would be to see if they correlate. However, there was little variation in driving speeds–hence, yaw rate, the independent variable–which prevented us from analyzing the dependency in this way. Hence, the Control, “normal” and “tp” groups were compared as follows. To evaluate the quantitative correspondence between pursuit and yaw rate, half of the measured average yaw rate value *during each detected individual pursuit* was subtracted from horizontal gaze–velocity on a “fixation–by–fixation” basis. This measure controls for individual differences is driving speed (and thus yaw rate), and, theoretically, should center around zero in the “normal” and control conditions (with perfect tracking OKN SP is equal to *½ yaw rate*), whereas for the “tp” condition the value should differ from zero by about *½ yaw rate*. For statistical analysis, the average difference between *gaze velocity* and *½ yaw–rate* was computed for each subject, over all trials within a condition (“normal”,”tp” and control).

We found that the zero-value–which would indicate equality of horizontal nystagmus speed and ½ yaw rate–is within the 95% confidence interval for the population mean for the “normal” condition [-0.68, 1.32], and for the control experiment [-0.79, 0.16], but not for the “tp” condition [4.57, 6.83]. The “normal” and “tp” conditions differed significantly on this measure (t(6) = 10.21, p = 5×10^−5^). The difference of “normal” and control did not reach significance (t(26) = 1.47, p = 0.15, 95% CI of the difference of independent samples, equal variances assumed [-1.55 0.26]). When the “normal” and control data are pooled, the 95% CI of the average values for this larger dataset was [-0.55, 0.29]. I.e. zero is still within the 95% CI, as predicted by the FP waypoint hypothesis.

Raw eye movement traces for Bend 2 for all valid trials and for all participants in Experiment 2 are given in the Supplement. The Control experiment raw traces are available in the supplement to [16].

The above results are thus consistent with drivers looking at waypoints on the future path, and this is true both in terms of the overall fixation distribution *and* the more informative and robust measure based on the direction and magnitude of the OKN SP, analyzed on a fixation–by–fixation basis. If the TP were indeed the predominant target of foveal gaze, gaze should be tightly clustered around it, and gaze velocity would be expected to remain reasonably close to zero (which means that the *gaze velocity – ½ vehicle yaw rate* should be distributed around – *½ vehicle yaw rate*, not around zero). This is what we find in the “tp” condition but *not* in the “normal” and control conditions, which we take to reflect constant–radius cornering in normal everyday driving.

## Discussion

The motivation for the present experiment was to evaluate two competing views on the nature of the OKN observed in previous studies:

1. the OKN SP is a naturally occurring strategy that serves a functional purpose and possibly top-down controlled, pursuit-like movement (according to the *future path waypoint tracking* hypothesis)
2. the OKN SP is an intrusive bottom-up reflex that cannot be suppressed, and therefore interferes with the top-down goal of fixating a travel point (according to the *tangent point hypothesis*)

We asked the subjects to alternate between “looking at the TP” while cornering vs.”driving as you would normally”, and the data from a previous experiment [16] was used as a control, i.e. to verify that the “normal” condition in fact corresponds to the naturalistic (uninstructed) gaze behavior of naïve subjects. When asked to fixate the tangent point drivers can do so, no OKN was observed in this condition. The present results therefore rule out interpretation (2) of the data from Experiment 1 – that is, that the OKN might be produced by an involuntary reflex even while “trying” to look at the TP. By asking the drivers to keep their eyes on the TP the OKN pattern generated to “normal” driving, we were thus able to show that the robust OKN in the normal condition is in fact not produced by an involuntary (unsuppressable) optokinetic reflex while “trying” to keep the gaze locked to the tangent point. This markedly different eye–movement behavior in the “normal” and “tp” conditions shows that the normal pattern does not correspond to a (voluntary) fixation of the TP with an OKR movement “capturing” gaze.

Optokinetic nystagmus was all but abolished in the “tp” condition (except for one participant who displayed clear far zone orientation and horizontal OKN even in the “tp” condition, S9, see Fig 7 and supplementary data in S1 Fig). In the “normal” condition OKN was for all intents and purposes indistinguishable from control condition. It was moreover consistent with waypoint tracking: when the gaze is fixed on a stationary point of fixation in the scene–such as a waypoint on the future path–the point of regard in the visual field tracks the point moving along the local visual flow which should have a horizontal speed of ½ vehicle yaw rate. We find the horizontal gaze speed during OKN SP quite nicely corresponds to this prediction.

### Relation to previous research

Tangent point models have been the prevailing explanation to eye movements observed during curve driving since [9]. Future path models have received some support, but mainly from simulator studies, and sometimes with explicit steering instruction, making the generalization to normal driving in the real world less than straightforward. The main body of evidence *for* tangent point orientation, however, cannot be considered as evidence *against* future path models because noise in gaze position measurement makes traditional AOI methods inconclusive due to the contiguity of tangent point and future path targets in the visual field causing the AOIs to overlap [14].

Kandil et al. [13], using a similar experimental design to ours (explicit gaze instructions), claim that “tangent point wins” over future path hypotheses in real–world driving. However, as noted, that experiment was hampered by weak data–analysis methods (see Introduction). Also, if by future path methods they are thinking of “gaze sampling” strategy instructed in their “gaze sampling” condition this is hardly surpising that the TP hypothesis wins over *this* hypothesis–for it is a highly artificial gaze pattern. Fixations tracking the same target location *for several seconds* does not reflect the normal pattern of optokinetic nystagmus in driving (where the OKN SP durations are typically around 200–400 ms; see [15],[16],[18], supplementary data in S1 Fig). Kandil et al. (ibid.) give no quantitative description of eye movement behavior to support their claims (merely reporting frequency tables for different “fixation targets” identified manually), and the fact that optokinetic nystagmus does indeed occur during curve negotiation casts some doubt as to whether their data analysis/visualization methods were in fact sensitive enough.

The present result is also in contrast to the Authié & Mestre [18] simulator study, in that in our on–road study the OKN SP is where one would expect it to be based on FP models, whereas in the Authié and Mestre [18] study OKN SP did *not* accurately follow focal flow at the estimated point of regard. In fact, it did not appear to accurately follow local or global flow at all: the correspondence between SP direction and flow direction in all the areas of interest around the gaze point was poor, the authors analyzed flow in AOI's of various radii: 0° (i.e. point of regard), 1°, 2°, 3°, 4°, 5°, 6°, 7°, entire viewing screen at 40°×49°, but none of these resulted in a good match between OKN SP orientation and flow orientation.

One possible reason for the difference is the visual stimulus: a bi–ocularly viewed screen with a restricted field of view *vs*. binocular viewing of a 3D scene with a larger field of view. Another difference is the task instruction: the participants in our experiments drove at a safe pace in real traffic, whereas the participants in the simulator study were asked to drive “as fast as possible without ever leaving the right lane”. Driving "as fast as possible" in a game environment (where judging speed and distance may be difficult, and the threats posed by leaving the road are less real), could lead to different low–level gaze behavior strategies from everyday driving. Another point to consider is that in *binocular* viewing, smooth pursuit follows flow of scene elements at the depth of the point of fixation, which means that in a real 3D environment optokinesis may also incorporate depth information by tracking retinal image elements with zero disparity, making stereo vision potentially relevant for steering (see discussion in [22]). We are not aware of studies where it would have been analyzed–either theoretically or experimentally–whether or not binocular disparity is actually used in driving.

Whatever the reason for the differing results–experimental instruction, stimulus properties etc.–a close correspondence between gaze velocity and yaw rate seems to characterize real driving ([15],[16], Experiment 2, present data).

## Conclusions

In summary, the results cast further evidence against a purely tangent point oriented model, and lend support for future path models postulating a waypoint on the future path that the driver identifies and tracks with an optokinetic pursuit movement [3],[5],[7].

One must be careful to steer clear of simplistic dichotomous conclusions, however. What we can say is that the predominant pattern in normal driving, OKN consistent with the visual flow in the far zone, is neither predicted by the tangent point hypothesis, nor observed in an experimental condition where the driver is instructed to keep their eyes on the tangent point. We cannot and do not claim that drivers never look at the tangent point in normal driving, nor that the data “disproves” all hypotheses about the usefulness of TP–based visual information. It is possible that steering is based on multiple visual reference points–producing more redundant and robust feedback–and that these reference points may not be the same in all situations (drivers of different experience and skill, curves of different visibility and radii, different curve phases).

It is clearly possible to successfully steer with gaze fixed to the tangent point. But unless instructed to do so, drivers do not behave in this way. Which raises the question: what cognitive processes underlie the visual orientation towards the far zone? What information is gleaned from the point of fixation, and what is the functional significance of the OKN SP? In particular, how is it related to steering control?

The most straightforward interpretation for the present results would be that gaze picks out waypoints on the desired future path. That is, the driver is “looking where (s)he wants to go”. This said, “wanting to go” somewhere is yet to be given a rigorous computational definition. FP waypoint models tend to be simple feedback models ([3],[5–7]) and do not describe in detail where, on the road ahead, the waypoint should be placed (and why), or what visual cues might be used. They are based on a feedback signal that presupposes an appropriately placed waypoint, but where the selection of waypoints is discussed at all, it is presumed to be a rather trivial affair—e.g. adjacent to or beyond the tangent point ([2],[4]). Thus, the present online-feedback waypoint models account for the slow pursuit phase of the OKN, but do not even account well for how the next waypoint target is selected (i.e. QP saccade planning)

We have here measured OKN on the constant–radius cornering phase of a long curve which is perhaps the simplest case of curvilinear path: little steering effort or advance motor planning is required (constant steering angle with minor corrections). It is even possible that steering information is in these conditions gleaned from peripheral vision (from various regions, including the TP area), and that looking at the road ahead serves a different purpose: to monitor surface roughness (i.e. watch out for puddles, potholes and other obstacles). The OKN would then be useful for stabilizing the retinal image for detailed visual analysis of surface pattern.

We have not analyzed the approach and entry to the curve which probably require more complex visual judgement, motor planning, and predictive control. Could TP *vs*. FP foveation even change during the curve driving sequence? The sequential organization of the curve–driving sequence is a topic that merits further study. Skilled action such as driving through bends always involves precisely timed action sequences that need to be planned some distance ahead for smooth performance. OKN/pursuit is observed particularly in the cornering and exit phases of a curve, and it is possible that different vision–action coupling may be at play when a driver judges the bend and initiated turn–in. It is possible that (more) tangent point foveation occurs during approach and turn–in. This, however, would be at present very challenging to study as during these phases the whole curve spans a very small area in the field of view–rendering AOI methods useless–and the OKN method cannot be used as the steering models do not make differential predictions in conditions where the car is not yet on a curvilinear trajectory.

It is possible that different targets and different strategies may be involved in different task phases (i.e. the tangent point could be used deciding when and by how much to turn the steering wheel). After all, it is typical in naturalistic tasks that sequential organization of the task is closely coupled to the patterns of eye movement behavior. What targets might be fixated at different phases of the curve, what information is extracted, and how that information is used to plan the future path (and at what subsequent point in time) remain important questions for future research. Our own position on the matter is that even the (arguably) simplest non-trivial steering task of steady state cornering cannot be explained mechanistically by a simple closed-loop model (at a bare minimum, some planning mechanism for generating the reference waypoint is needed to determine the feedback error is required), less so the more challenging tasks of “judging” a bend during approach and initiating a well-timed turn-in response of appropriate amplitude.

For future theoretical development, this would mean that the mathematical sophistication of the models will have to be of a higher degree, integrating much more visual information from the scene than a single steering point and incorporating predictive state estimation and motor planning (and not just for steering, speed selection but oculomotor control as well). Such more general models will likely need to be founded on advanced computational methods such as machine learning and advanced statistical modeling, as opposed to the simple closed-loop feedback idea—borrowed from control theory—characteristic of almost all current visual steering models in the tangent point/future path literature [12].

## Supporting Information

S1 Fig Supplementary results showing raw eye movement data, and algorithmic OKN SP identification results for Bend two for all valid trials for all participants in Experiment 2 (NB. Similar plots for the Control condition are available in the supplement to Lappi, Pekkanen & Itkonen (2013), doi: 10.1371/journal.pone.0068326.
(PDF)

## References

[1] Donges E. A two-level model of driver steering behavior. Hum Factors, 1978; 20: 691–707.

[2] Boer ER. Tangent point oriented curve negotiation. Intelligent Vehicles Symposium 1996, Proceedings of the 1996 IEEE, 1996; 7–12.

[3] Wann JP, Swapp D. Why you should look where you are going. Nat Neurosci, 2000; 3: 647–648. 10862695 10.1038/76602

[4] Wann JP, Land MF. Steering with or without the flow: is the retrieval of heading necessary? Trends Cogn Sci, 2000; 4: 319–324. 10904256 10.1016/s1364-6613(00)01513-8

[5] Wann JP, Wilkie RW. How do we control high speed steering? In: Vaina LM, Beardsley SA, Rushton SK editors: Optic Flow and Beyond. Kluwer Academic Publishers Norwell, MA, USA; 2004 p. 371–389.

[6] Salvucci D, Gray R. A two-point visual control model of steering. Perception, 2004; 33: 1233–1248. 15693668 10.1068/p5343

[7] Wilkie RW, Wann JP, Allison R. Active Gaze, visual look-ahead, and locomotor control. J Exp Psych: Hum Perc Perf, 2008; 34: 1150–1164. 10.1037/0096-1523.34.5.1150 18823202

[8] Raviv D, Herman M. A new approach to vision and control for road following. Proceedings of the IEEE workshop on visual motion; 10.1109/WVM.1991.212804

[9] Land MF, Lee D. Where we look when we steer. Nature, 1994; 369: 742–744. 8008066 10.1038/369742a0

[10] Land MF. The visual control of steering In: Harris L.R. & Jenkin M. (eds.): Vision and action. Cambridge university press: Cambridge, 1998 p. 163–180.

[11] Authié CN & Mestre DR. Path curvature discrimination: dependence on gaze direction and optical flow speed. PLoS ONE, 2012; 7, e31479 10.1371/journal.pone.0031479 22393363 PMC3290598

[12] Lappi O (2014) Future path and tangent point models in the visual control of locomotion in curve driving. J Vis, 2014; 14: 1–22. 10.1167/14.12.21 25761280

[13] Kandil F, Rotter A, Lappe M. Driving is smoother and more stable when using the tangent point. J Vis, 2009; 9: 1–11. 10.1167/9.1.11 19271881

[14] Lappi O, Lehtonen E, Pekkanen J, Itkonen TH. Beyond the tangent point–gaze targets in naturalistic driving. J Vis, 2013; 13: 11–18. 10.1167/13.13.11 24222181

[15] Lappi O, Lehtonen E. Eye-movements in real curve driving: pursuit-like optokinesis in vehicle frame of reference, stability in an allocentric reference coordinate system. J Eye Mov Res, 2013; 6: 1–13.

[16] Lappi O, Pekkanen J, Itkonen T. Pursuit eye-movements in curve driving differentiate between future path and tangent point models. PLOS ONE, 2013; 8, e68326 10.1371/journal.pone.0068326 23894300 PMC3718775

[17] Kim N-G, Turvey MT. Eye movements and a rule for perceiving direction of heading. Ecol Psych, 1999; 11: 233–248.

[18] Authié CN, Mestre DR. Optokinetic nystagmus is elicited by curvilinear optic flow during high speed curve driving, Vis Res, 2010; 51: 1791–1800. 10.1016/j.visres.2011.06.010 21704061

[19] Jackson B, Scargle JD, Barnes D, Arabhi S, Alt A, Giomousis P et al An algorithm for optimal partitioning of data on an interval. IEEE Signal Proc Letters, 2005; 12: 105–108.

[20] Killick R, Fearnhead P, Eckley I. Optimal detection of changepoints with a linear computational cost. Journal of the American Statistical Association, 2012; 107 (500), 1590–1598.

[21] Bradski G. The OpenCV Library. Dr. Dobb's Journal of Software Tools, 2000.

[22] Lappi O. Eyes on the road–the visual control of locomotion in curve driving (Studies in Cognitive Science 05/2013). PhD dissertation. University of Helsinki, 2013.

