# Correction: Driver Gaze Behavior Is Different in Normal Curve Driving and when Looking at the Tangent Point

**DOI:** 10.1371/journal.pone.0139410

**Published:** 2015-09-24

**Authors:** The PLOS ONE Staff

There are errors in the Funding section. The publisher apologizes for the errors. The correct funding information is as follows: Mr. Jami Pekkanen received funding from a personal grant from Finnish Cultural Foundation (grant no. 00140724). Mr. Jami Pekkanen and Mr. Teemu Itkonen were employed in MULSIMCO project funded by the Finnish Academy (www.aka.fi, project no. 1279905 PI prof. Heikki Summala). The funders had no role in study design, data collection and analysis, decision to publish, or preparation of the manuscript.
