# Supplementary material for: Driver Gaze Behavior Is Different in Normal Curve Driving and when Looking at the Tangent Point
Source: PLoS One. 2015 Aug 19;10(8):e0135505. doi: 10.1371/journal.pone.0135505 (PMC4546002; doi:10.1371/journal.pone.0135505)
Supplement: S1 Fig — Similar plots for the Control condition are available in the supplement to Lappi, Pekkanen & Itkonen (2013), doi: 10.1371/journal.pone.0068326. (PDF) [file pone.0135505.s001.pdf]

Single trial data for Bend 2

Raw horizontal gaze direction estimate, from the eye tracker, relative to the vehicle frame of reference (0° = straight ahead), is plotted on the y axis Upwards it to the right (the direction of bend curvature) . Time (in secondes, from the beginning of the experiment) is on the x axis. The red linear segments are the pursuits identified by the pursuit detection algorthm.

Plotted are all valid trials in Bend 2 from Experiment 2. (This is the same bend that was analysed in Experiment 1. Thhus, to compare single trial data in the Normal and Control conditions, please refer to Supplementary figures in Lappi O, Pekkanen J, Itkonen TH (2013) Pursuit Eye-Movements in Curve Driving Differentiate between Future Path and Tangent Point Models. PLoS ONE 8(7): e68326. doi:10.1371/journal.pone.0068326)

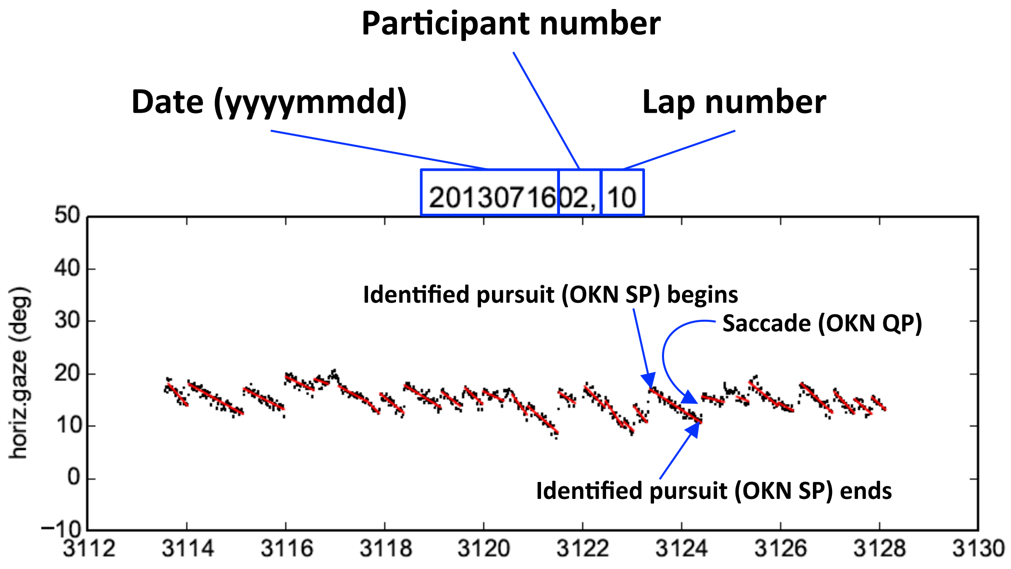

SUPPLEMENTARY FIGURE S1

SUBJECT 2 NORMAL CONDITION BEND 2

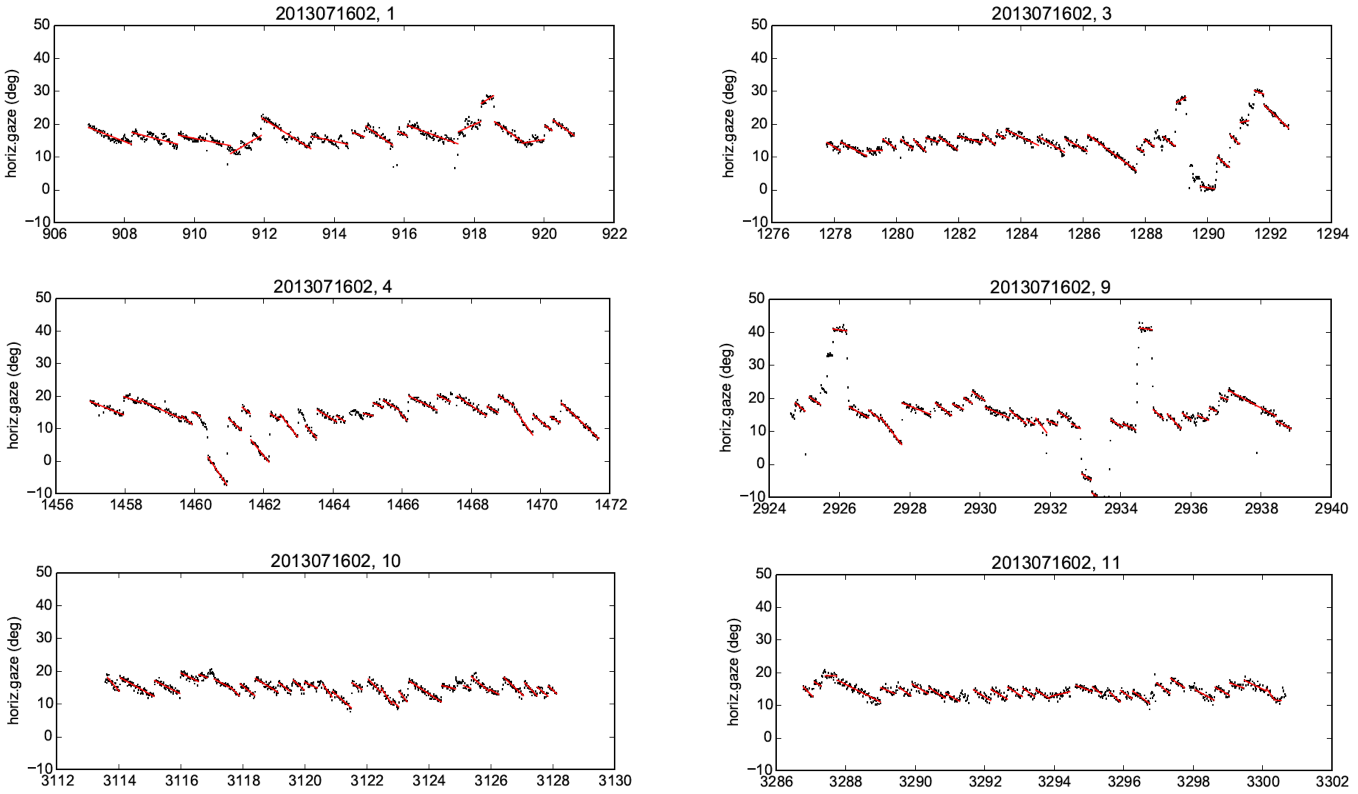

SUPPLEMENTARY FIGURE S2

SUBJECT 2 TP CONDITION BEND 2

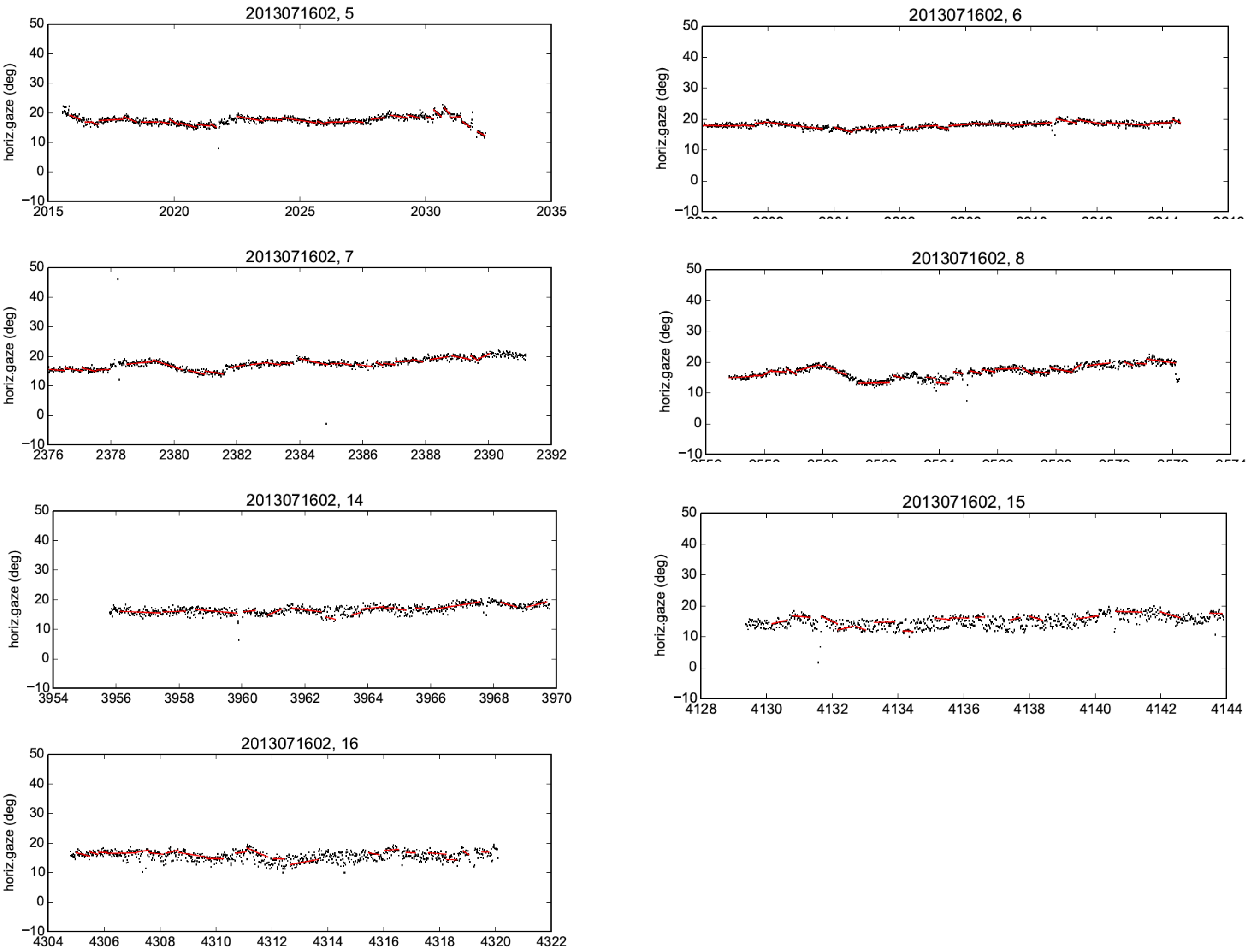

SUPPLEMENTARY FIGURE S3

SUBJECT 4 NORMAL CONDITION BEND 2

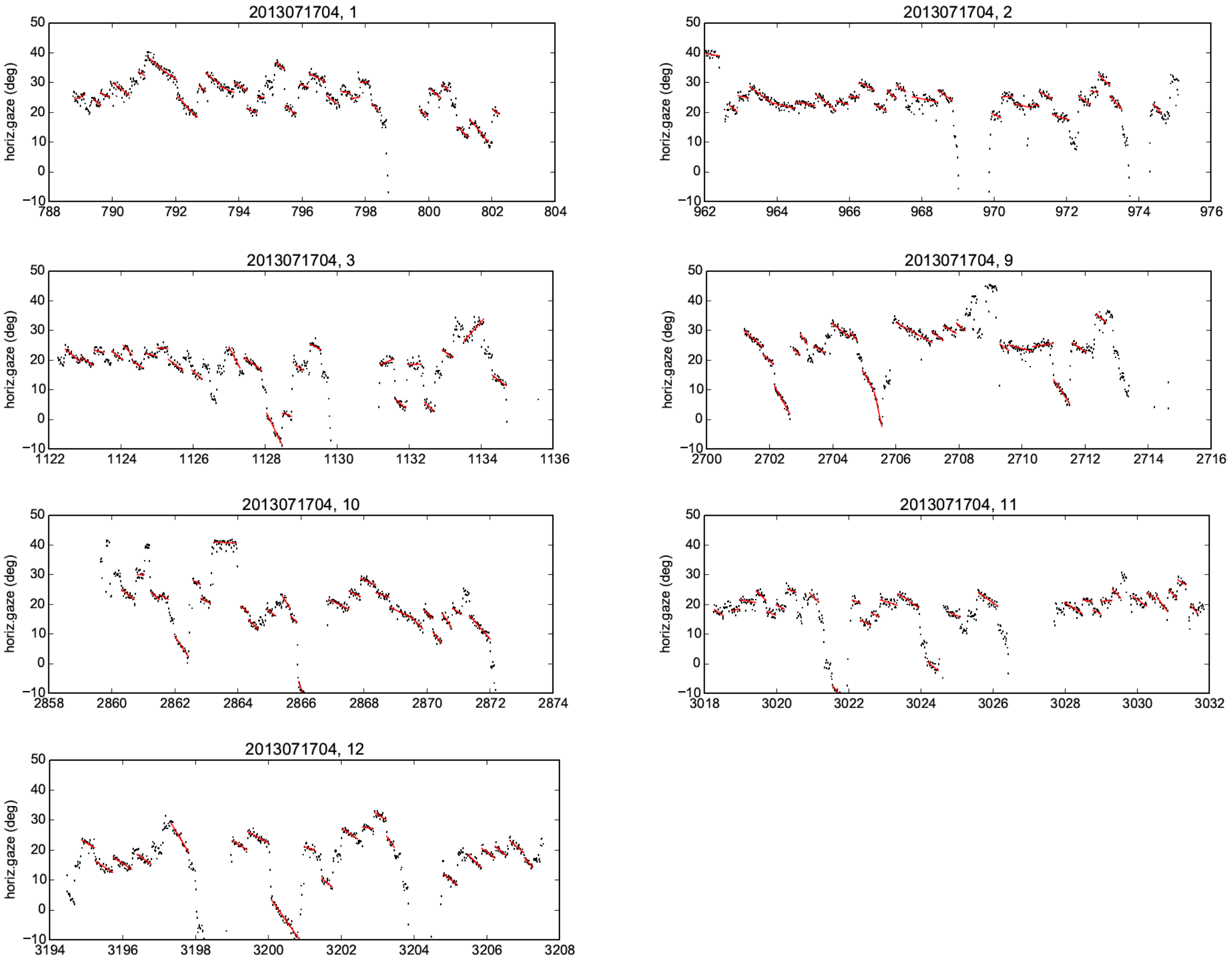

SUPPLEMENTARY FIGURE S4

SUBJECT 4 TP CONDITION BEND 2

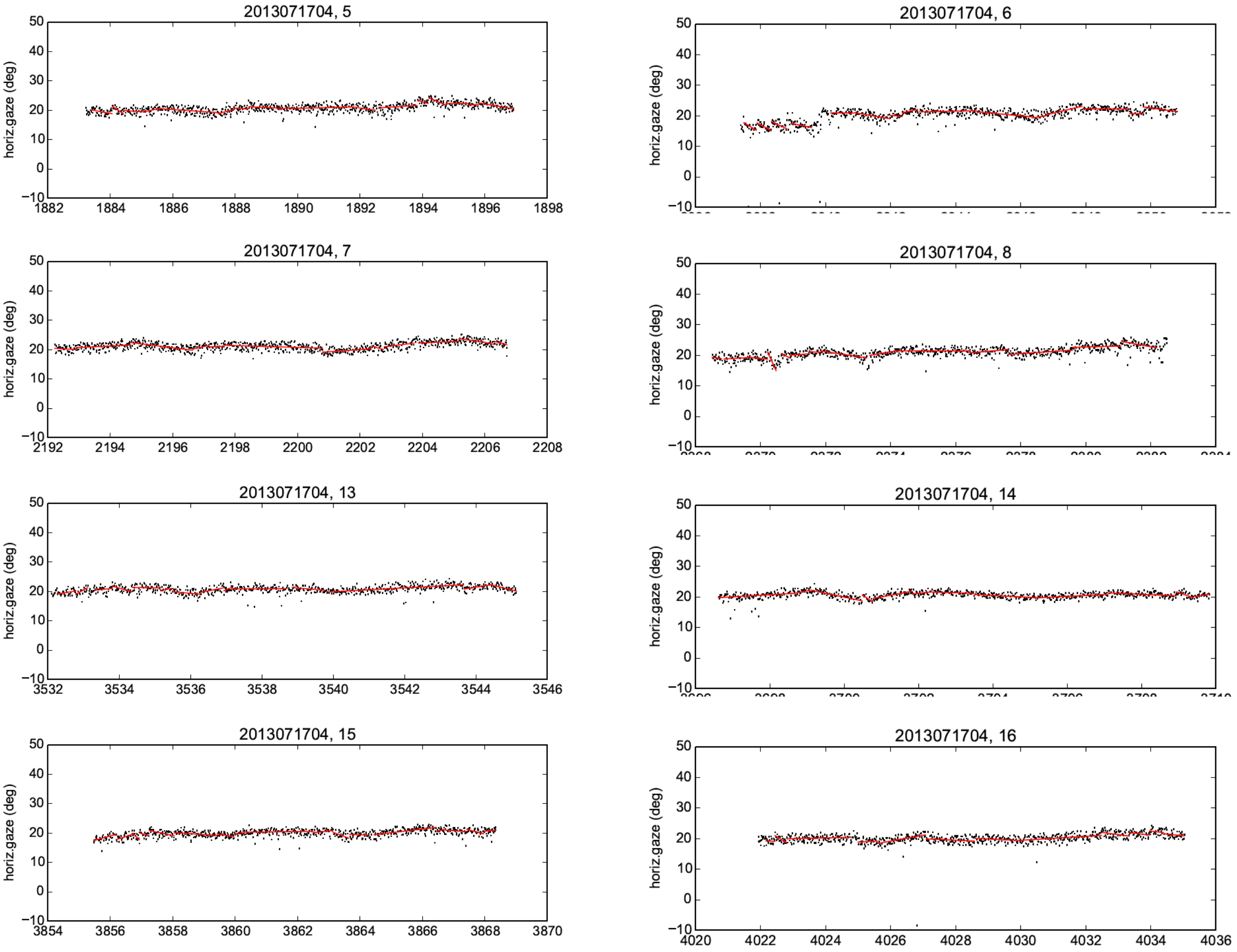

SUPPLEMENTARY FIGURE S5

SUBJECT 5 NORMAL CONDITION BEND 2

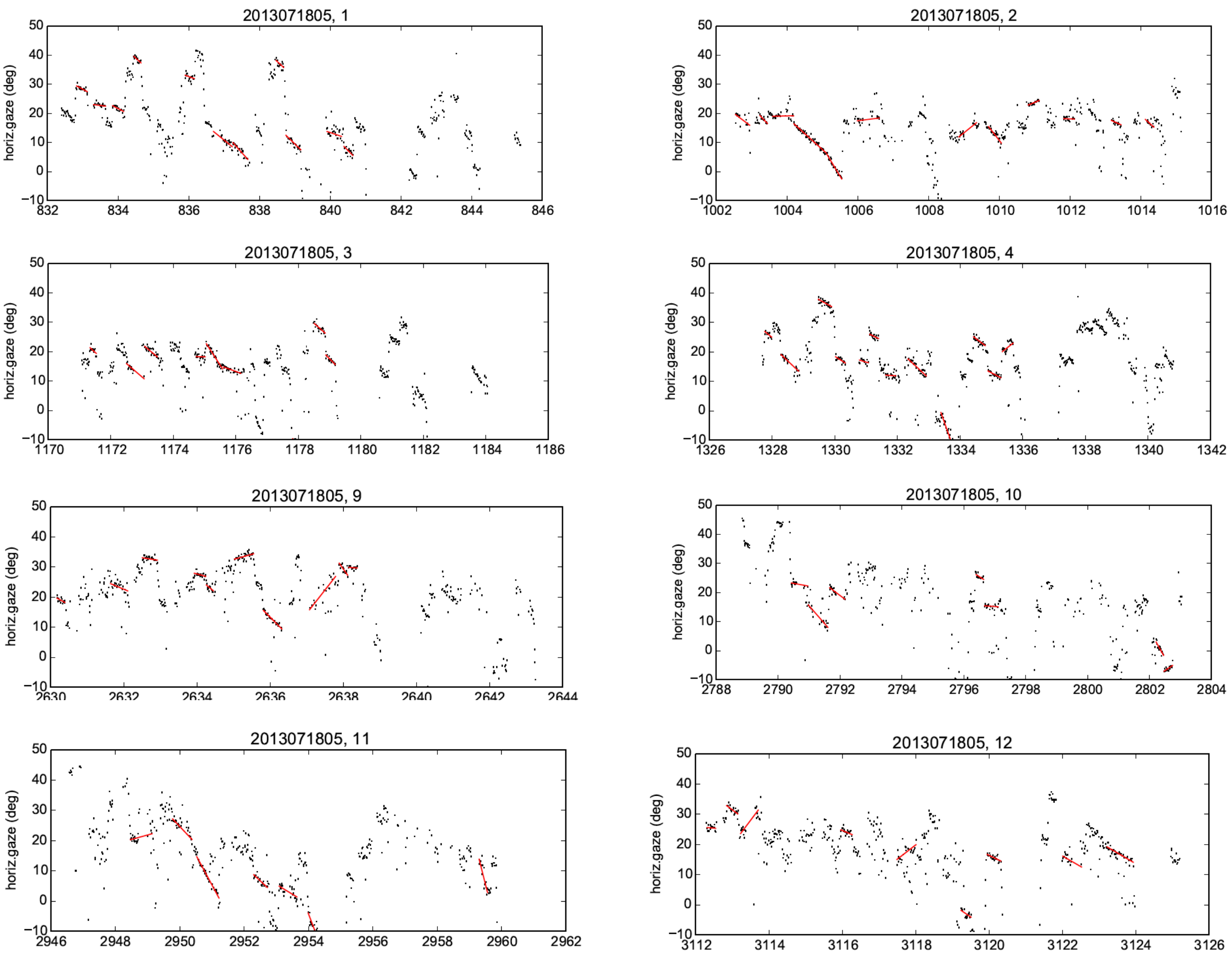

SUPPLEMENTARY FIGURE S6

SUBJECT 5 TP CONDITION BEND 2

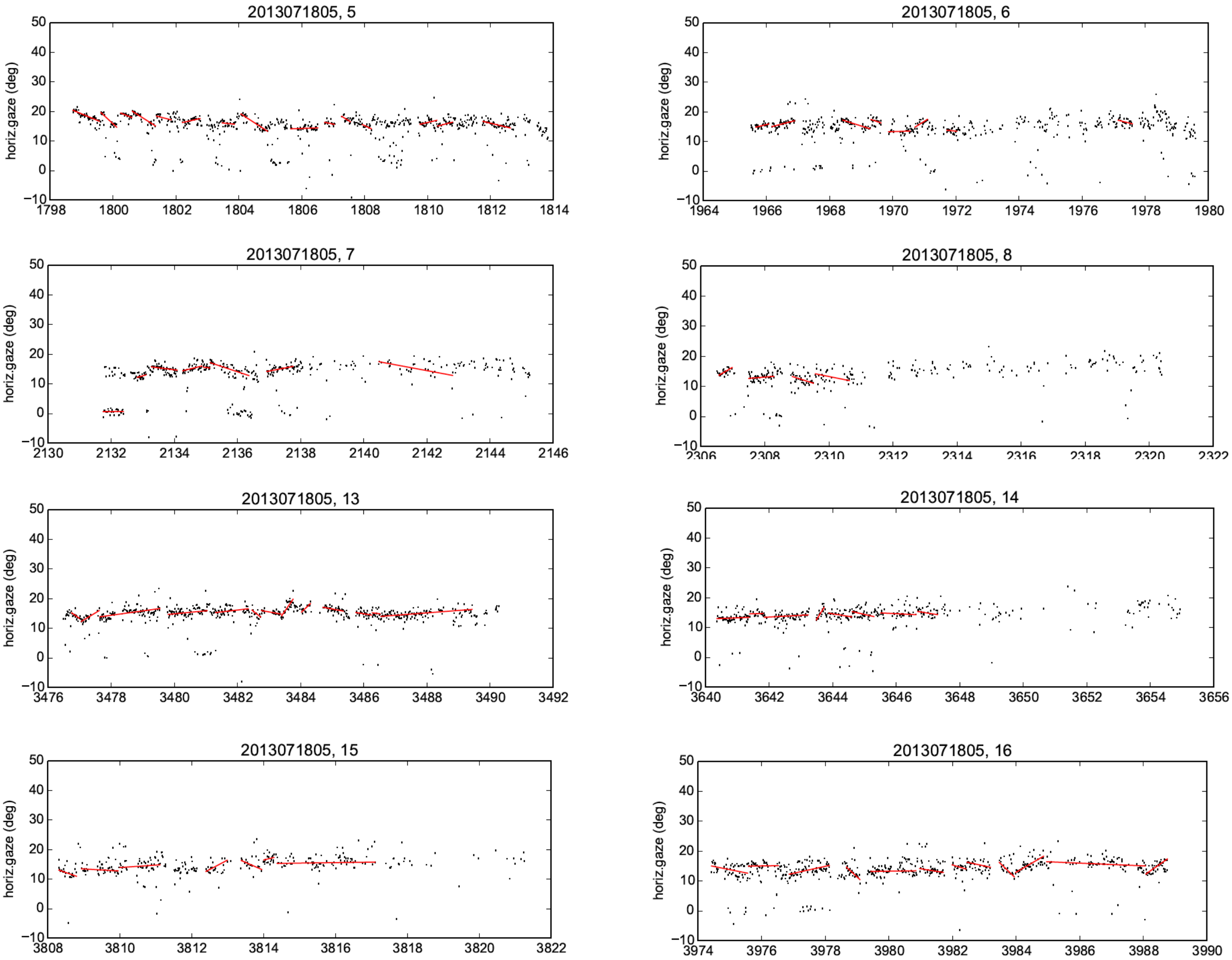

SUPPLEMENTARY FIGURE S7

SUBJECT 6 NORMAL CONDITION BEND 2

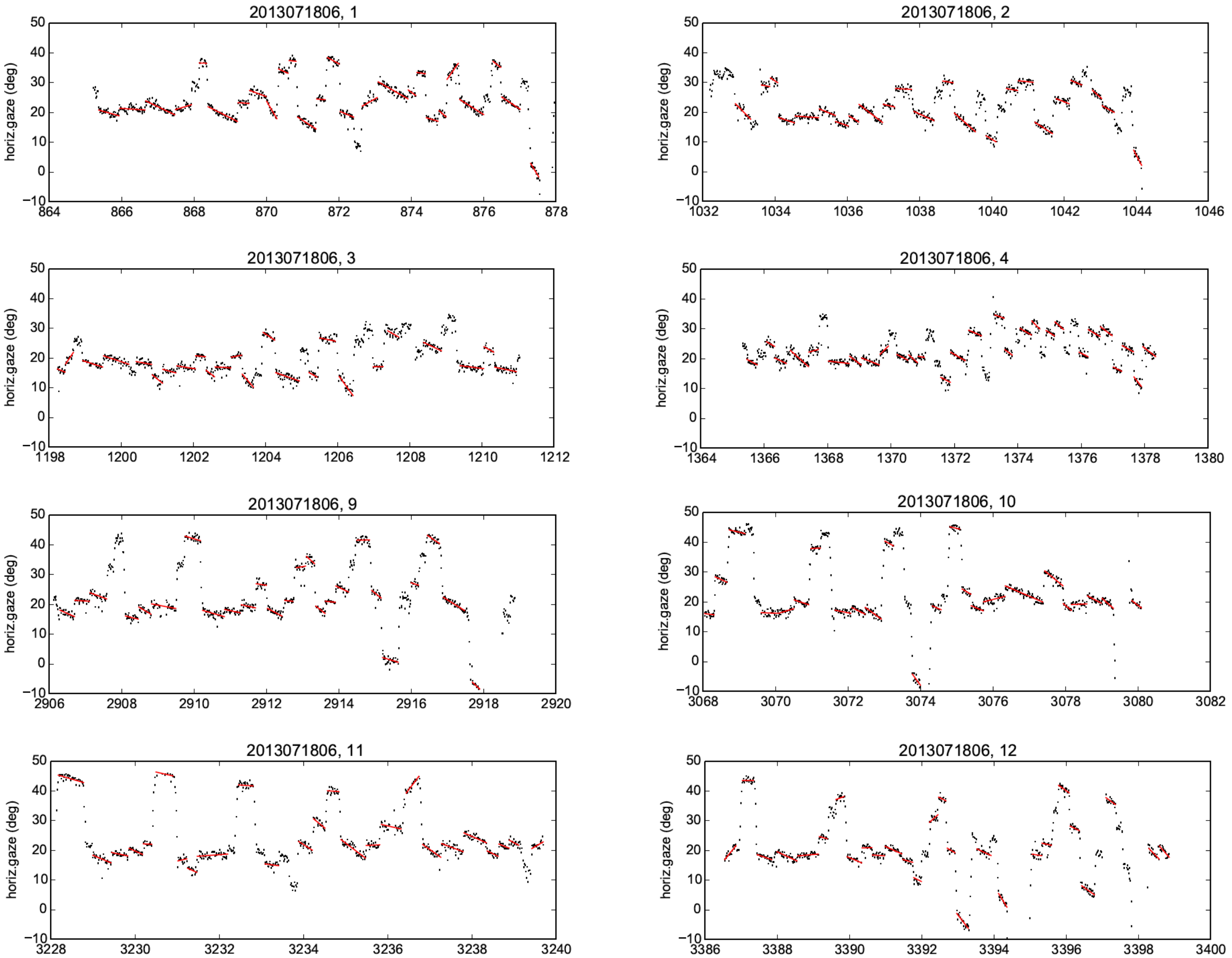

SUPPLEMENTARY FIGURE S8

SUBJECT 6 TP CONDITION BEND 2

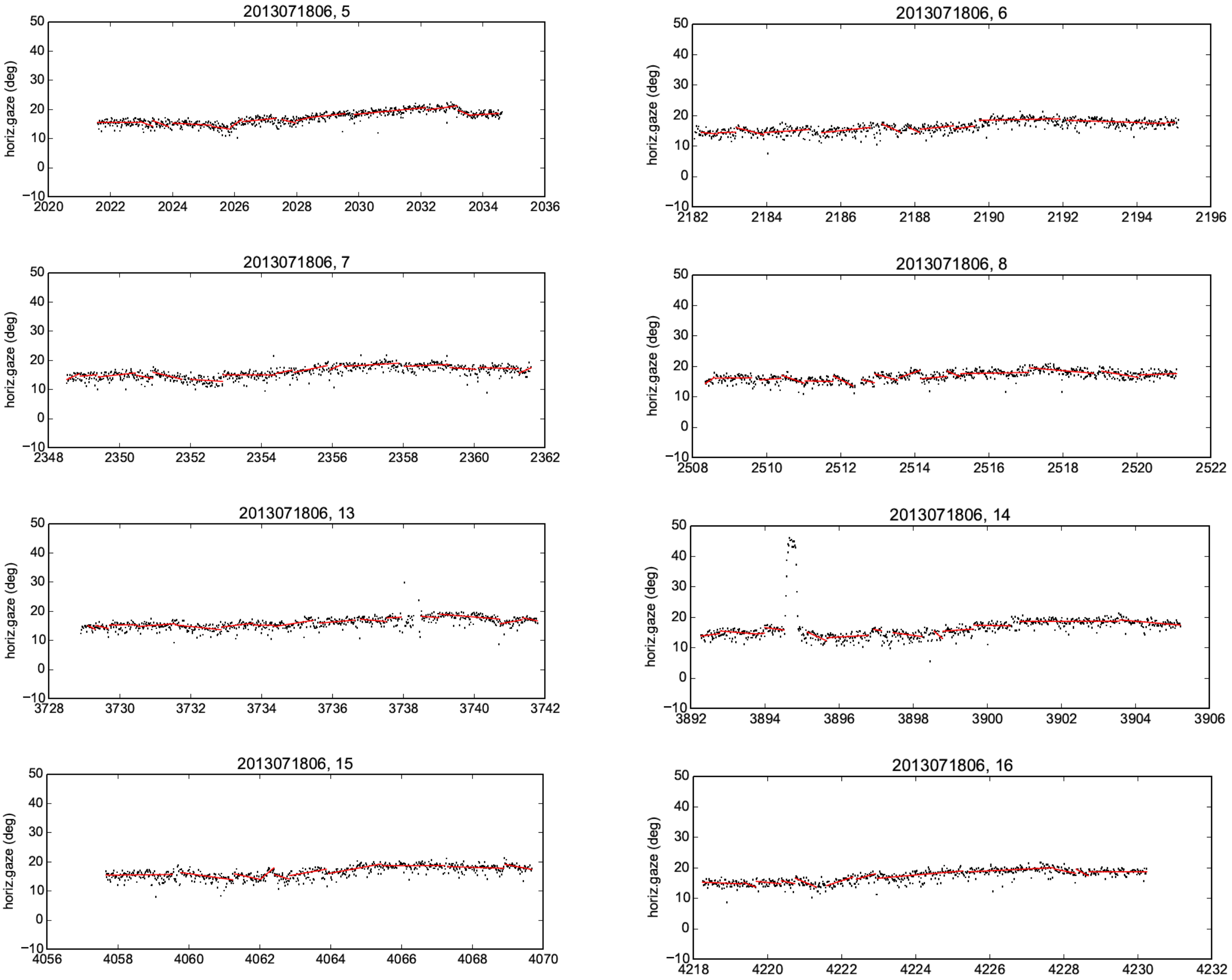

SUPPLEMENTARY FIGURE S9

SUBJECT 8 NORMAL CONDITION BEND 2

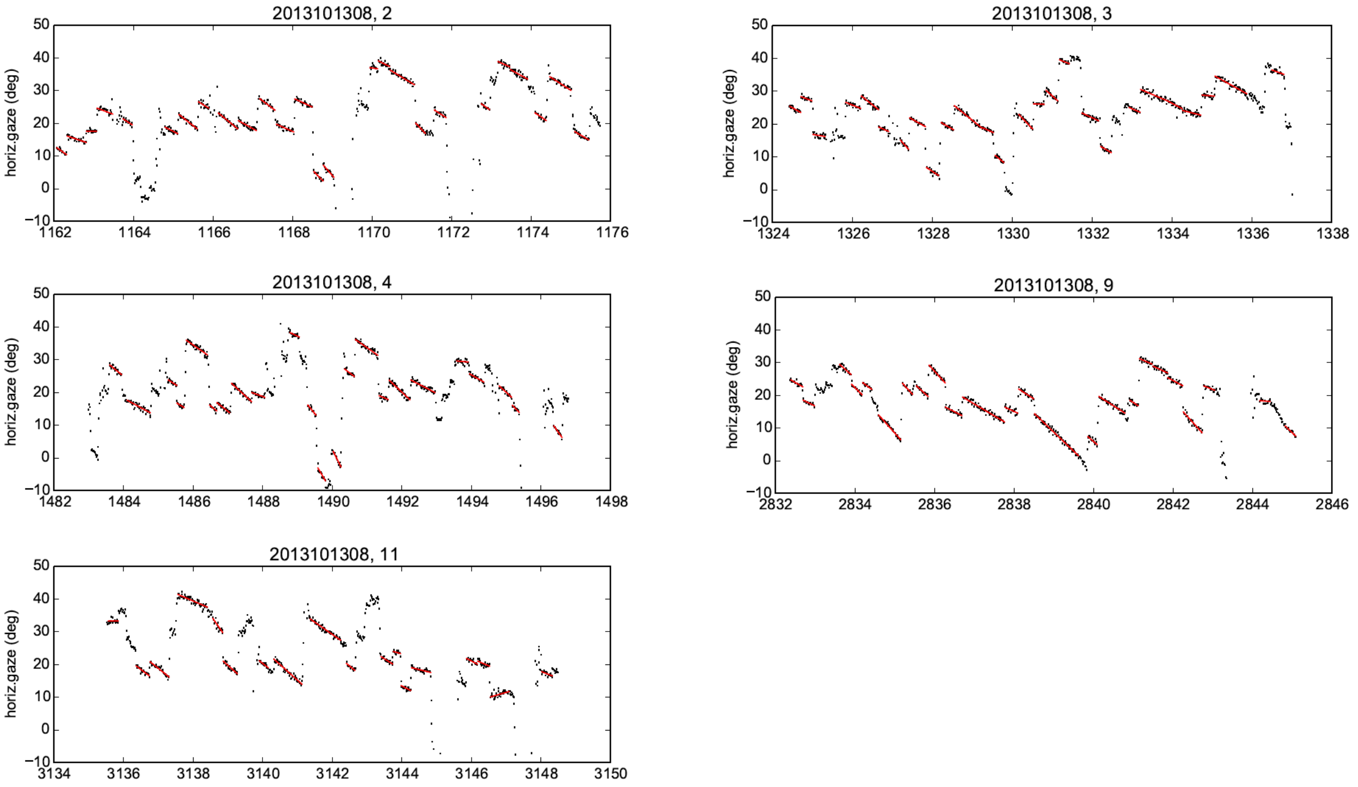

SUPPLEMENTARY FIGURE S10

SUBJECT 8 TP CONDITION BEND 2

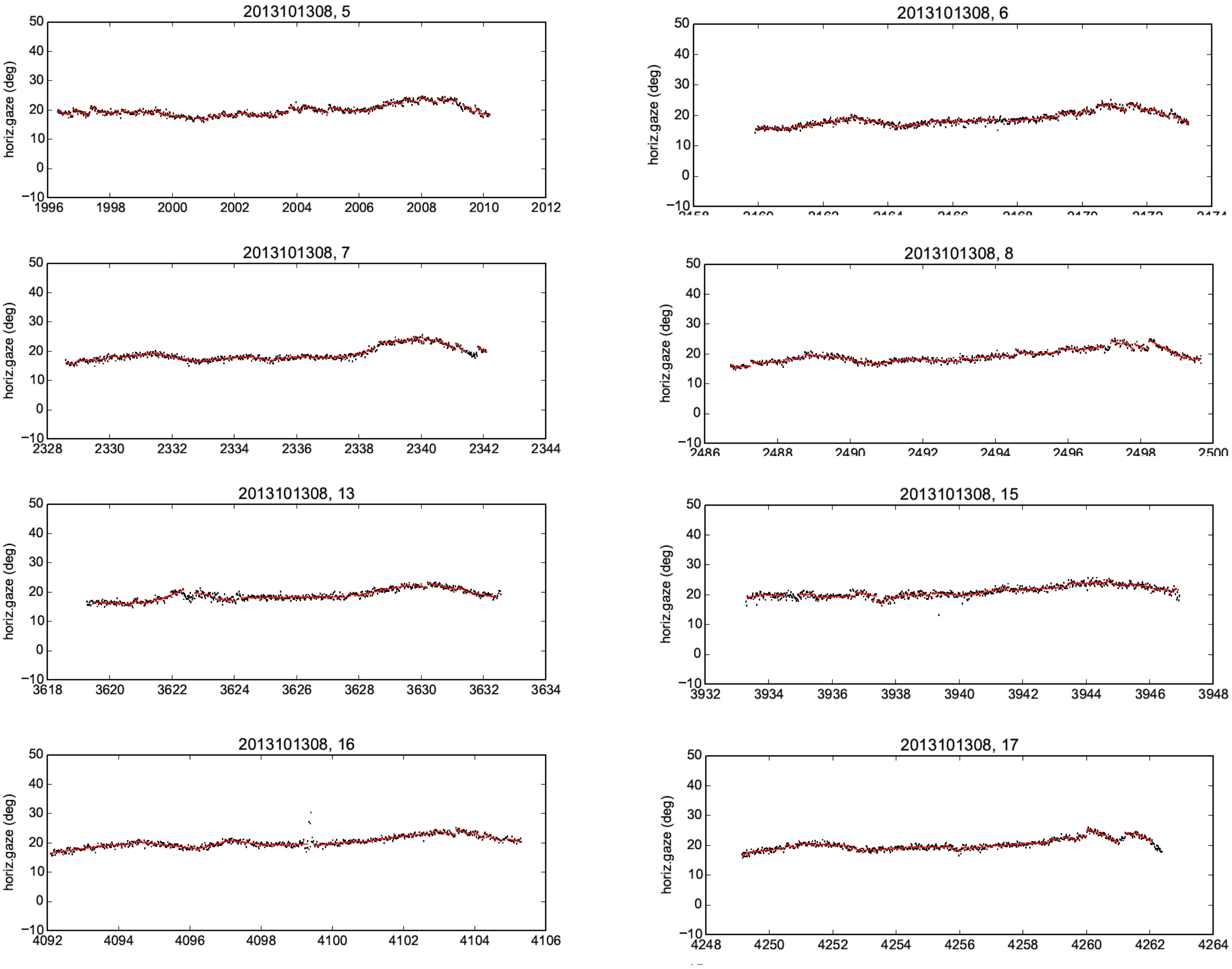

SUPPLEMENTARY FIGURE S11

SUBJECT 9 NORMAL CONDITION BEND 2

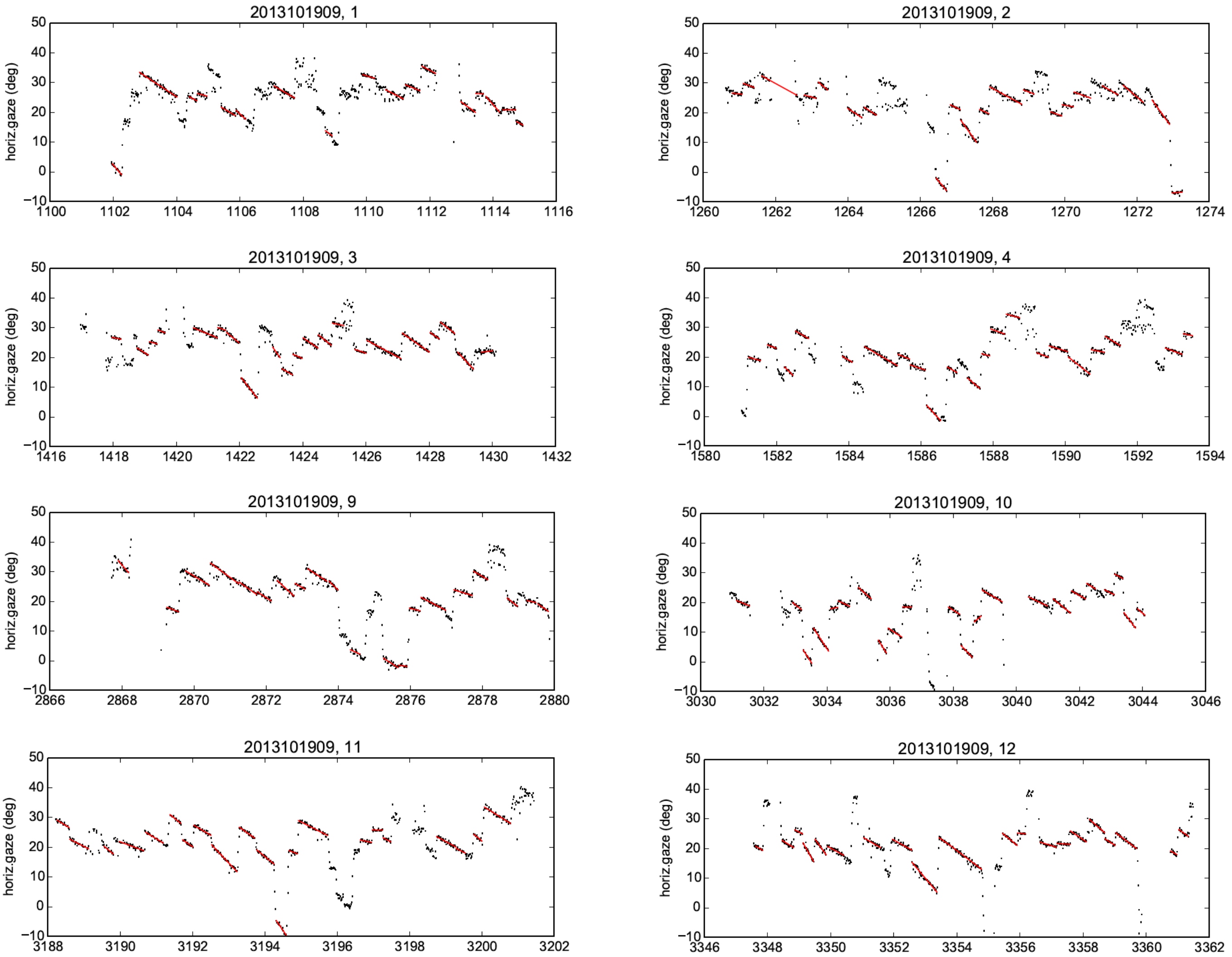

SUPPLEMENTARY FIGURE S12

SUBJECT 9 TP CONDITION BEND 2

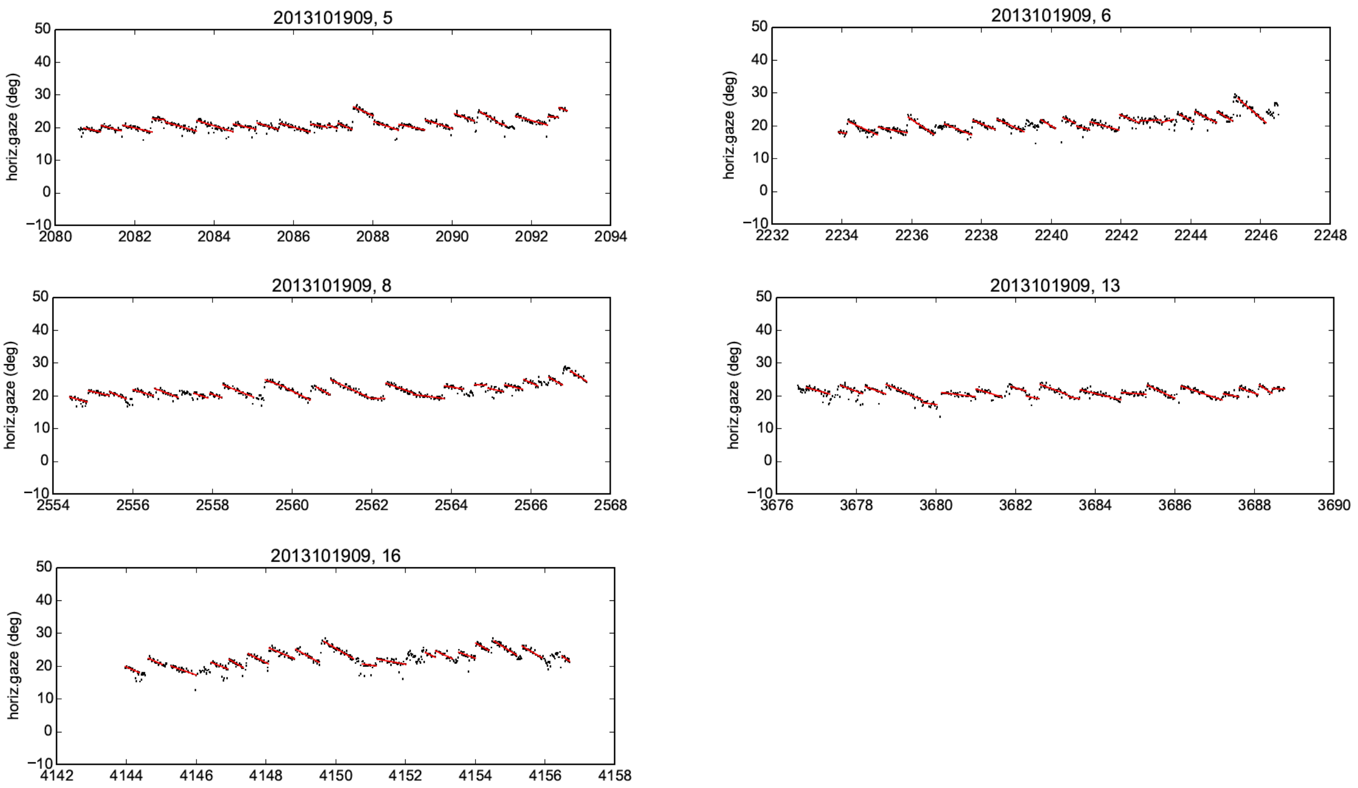

SUPPLEMENTARY FIGURE S13

SUBJECT 10 NORMAL CONDITION BEND 2

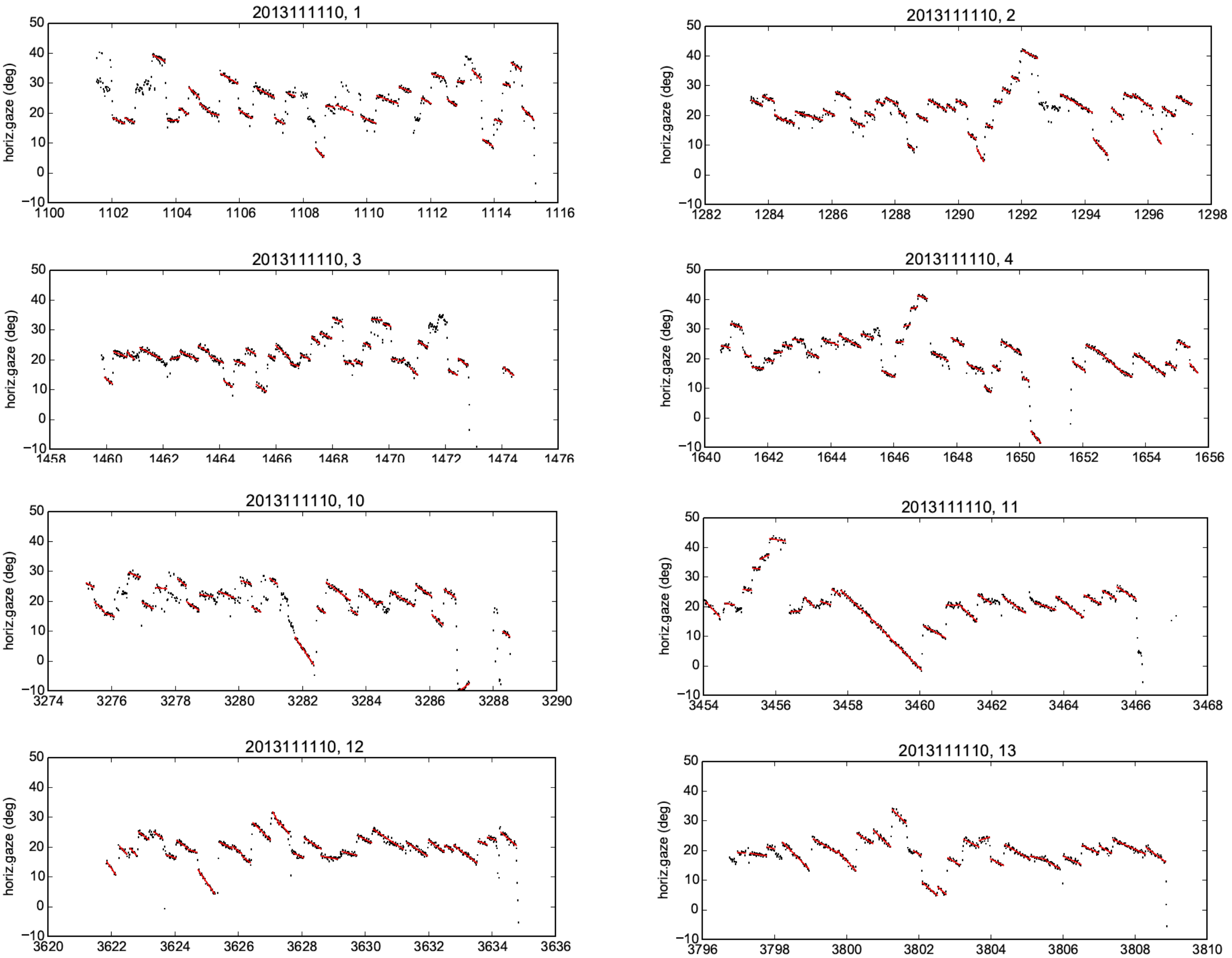

SUPPLEMENTARY FIGURE S14

SUBJECT 10 TP CONDITION BEND 2

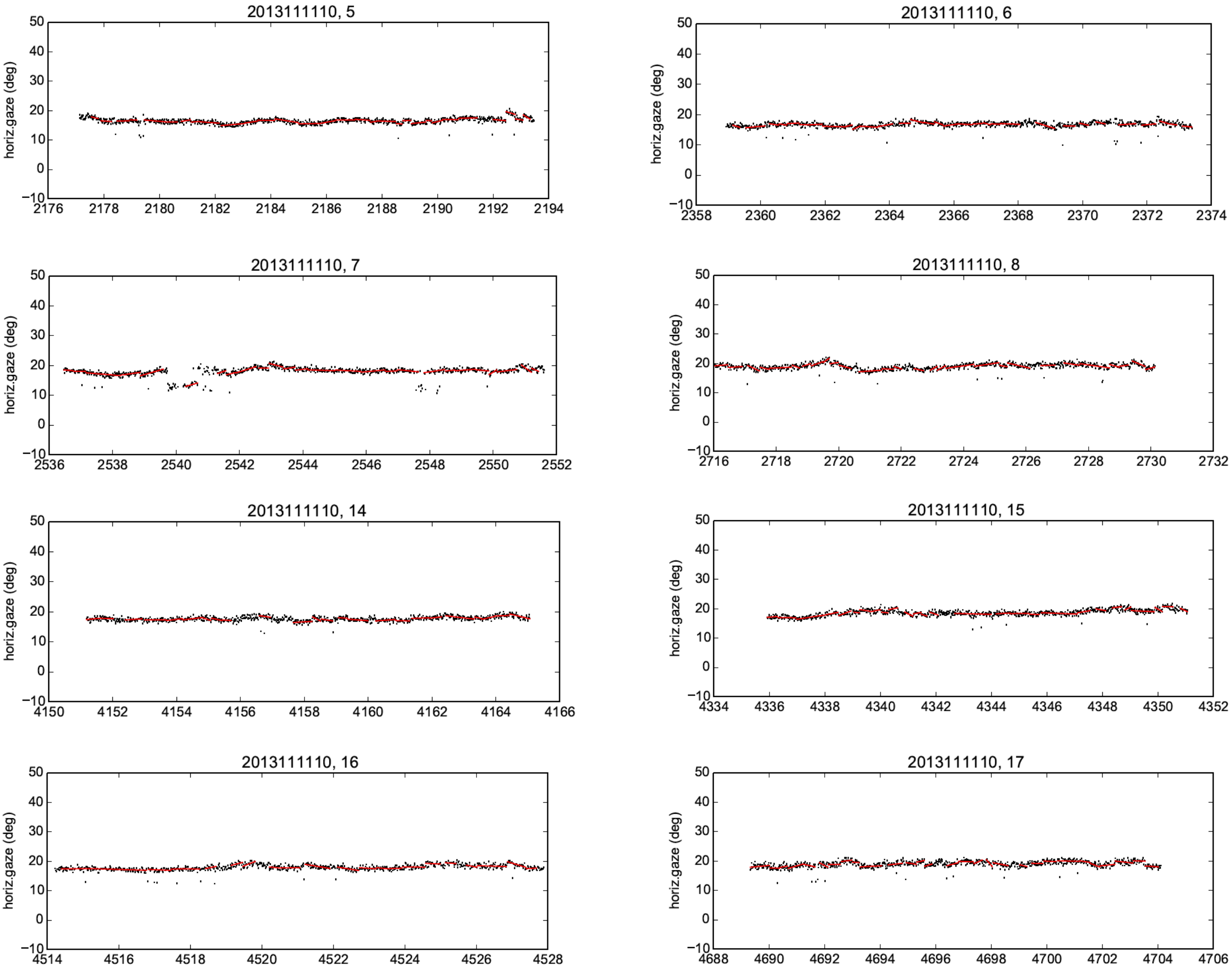

SUPPLEMENTARY FIGURE S15
